# Small RNAs derived from tRNAs and rRNAs are highly enriched in exosomes from both old and new world Leishmania providing evidence for conserved exosomal RNA Packaging

**DOI:** 10.1186/s12864-015-1260-7

**Published:** 2015-03-05

**Authors:** Ulrike Lambertz, Mariana E Oviedo Ovando, Elton JR Vasconcelos, Peter J Unrau, Peter J Myler, Neil E Reiner

**Affiliations:** Departments of Medicine, Microbiology and Immunology, University of British Columbia, Vancouver, BC Canada; Department of Molecular Biology and Biochemistry, Simon Fraser University, Burnaby, BC Canada; Seattle Biomedical Research Institute, Seattle, WA USA; Departments of Global Health and Biomedical Informatics & Medical Education, University of Washington, Washington, WA USA

**Keywords:** Leishmania, Exosomes, Shuttle RNA, Small non-coding RNA, tRNA-derived small RNA

## Abstract

**Background:**

Leishmania use exosomes to communicate with their mammalian hosts and these secreted vesicles appear to contribute to pathogenesis by delivering protein virulence factors to macrophages. In other eukaryotes, exosomes were found to carry RNA cargo, such as mRNAs and small non-coding RNAs, capable of altering recipient cell phenotype. Whether leishmania exosomes also contain RNAs which they are able to deliver to bystander cells is not known. Here, we show that leishmania exosomes indeed contain RNAs and compare and contrast the RNA content of exosomes released by *Leishmania donovani* and *Leishmania braziliensis*.

**Results:**

We purified RNA from exosomes collected from axenic amastigote culture supernatant and found that when compared with total leishmania RNA, exosomes mainly contained short RNA sequences. Exosomes with intact membranes were capable of protecting their RNA cargo from degradation by RNase. Moreover, exosome RNA cargo was delivered to host cell cytoplasm *in vitro*. Sequencing of exosomal RNA indicated that the majority of cargo sequences were derived from non-coding RNA species such as rRNA and tRNA. In depth analysis revealed the presence of tRNA-derived small RNAs, a novel RNA type with suspected regulatory functions. Northern blotting confirmed the specific and selective enrichment of tRNA-derived small RNAs in exosomes. We also identified a number of novel transcripts, which appeared to be specifically enriched in exosomes compared to total cell RNA. In addition, we observed the presence of sequences mapping to siRNA-coding regions in *L. braziliensis* , but not in *L. donovani* exosomes.

**Conclusions:**

These results show that leishmania exosomes are selectively and specifically enriched in small RNAs derived almost exclusively from non-coding RNAs. These exosomes are competent to deliver their cargo of novel, potential small regulatory RNAs to macrophages where they may influence parasite-host cell interactions. The remarkably high degree of congruence in exosomal RNA content between *L. donovani* and *L. braziliensis*, argues for the presence of a conserved mechanism for exosomal RNA packaging in leishmania. These findings open up a new avenue of research on non-canonical, small RNA pathways in this trypanosomatid, which may elucidate pathogenesis and identify novel therapeutic approaches.

**Electronic supplementary material:**

The online version of this article (doi:10.1186/s12864-015-1260-7) contains supplementary material, which is available to authorized users.

## Background

Protozoan parasites of the genus *Leishmania* are highly endemic to tropical and sub-tropical regions of the world. They are transmitted to humans and other mammals by sandfly vectors that inject the flagellated, promastigote life cycle stage of leishmania into the dermis of the host while taking a blood meal. After innoculation, promastigotes are engulfed by host mononuclear phagocytes either directly or indirectly as cargo of apoptotic neutrophils [1]. Following their ingestion by host cells, promastigotes take up residence in the phagolysosome, where they transform into amastigotes and undergo cell proliferation. Depending on the infecting leishmania species, disease manifestations and symptoms can vary widely from mild self-healing cutaneous lesions to lethal visceral disease. The two species that are the focus of the present study, *Leishmania donovani* and *Leishmania braziliensis*, cause visceral and mucocutaneous leishmaniasis, respectively. While the former is naturally the more serious threat as it can lead to death if left untreated, the latter can have an extremely high impact on the affected individual due to debilitating and disfiguring destruction of critical soft tissue structures.

The current paucity of effective and well tolerated drug treatments and even more so, the lack of highly efficacious, well standardized and widely available vaccination strategies can be attributed at least in part to the gap of knowledge about the intricate interplay between leishmania and host macrophages. Macrophages are universal key players in both innate and adaptive immune responses. Their primary function is to engulf and digest prey, whether pathogen or debris from cellular turnover, which makes their intracellular environment very nutrient-rich. Leishmania exploits these macrophage characteristics in a very sophisticated manner: it lets the phagocyte ingest it, and then uses the cell as its safe nursery, where it scavenges nutrients and replicates while remaining unrecognized by other immune cells. The mechanisms by which leishmania manages to survive within these potent immune cells are just starting to be elucidated. One key strategy employed by leishmania appears to be the prevention of macrophage activation, a step that is crucial to induce macrophage digestion and killing functions [2-4]. At the same time, leishmania are resistant to the harsh conditions of the acidifying phagolysosome [5].

In principle, there are two categories of molecules –surface associated and secreted- made available by leishmania to communicate with the host and turn on and off macrophage cellular functions. Regarding secreted molecules, our group has recently discovered that leishmania use a non-classical secretion mechanism to export a majority of their secreted proteins, which involves the release of small vesicles called exosomes [6,7].

Exosomes are 50–100 nanometre-sized membrane vesicles secreted by a variety of single- as well as multi-cellular eukaryotic organisms. They are distinct from membrane microvesicles, which are produced by blebbing, since their release occurs through fusion of multivesicular bodies from the endocytic/exocytic pathway with the plasma membrane of the cell [8]. Extracellular vesicles such as microvesicles and exosomes had long been considered to be simply cellular garbage bags. Only recently has the release of specific cargo within vesicles, as well as their uptake and effects on recipient cells, been appreciated to represent important biological events. Extracellular vesicle release has also been documented in the context of infection, where the vesicles were shown to contain both host and pathogen-derived antigens and virulence factors (reviewed in [9]). Extracellular vesicles containing pathogen derived factors, may be released either by infected cells, as has been shown following infection with Eppstein-Barr virus, mycobacteria, toxoplasma or plasmodia [10-13], or released by the pathogen directly, e.g. mycobacteria, cryptococci, trypanosoma and leishmania [7,14-17].

Importantly, in our studies, *L. donovani* exosomes and exosomal proteins were detected in the cytosolic compartment of infected macrophages [7]. Moreover, we showed that *L. donovani* exosomes can modulate mononuclear cell phenotypes *in vitro*, rendering them anti-inflammatory by specifically inhibiting cytokine production. Studies with C57Bl/6 and Balb/c mice provided evidence that treatment with exosomes from *L. donovani* as well as *Leishmania major* prior to infection exacerbated disease *in vivo* [18]. These findings have fundamentally transformed our understanding of how leishmania are able to communicate with the host. Two other studies have since supported a role for exosomes in leishmania pathogenesis. In the first one, the authors showed that the metalloprotease GP63 delivered by *L. donovani* exosomes cleaved the microRNA (miRNA) processing nuclease Dicer 1 in murine hepatocytes, resulting in downregulation of microRNA-122 expression, lowering of serum cholesterol and enhancement of murine liver infection [19]. In a second study, another group looking at *L. major* exosomes reported that the vesicles globally affected macrophage gene expression, which was in part GP63-dependent [20]. In summary, these results make a strong case for the importance of exosomes in leishmania pathogenesis.

In addition to their protein cargo, exosomes and microvesicles were recently shown to be carriers of nucleic acids in the form of RNA. This observation was first made in mast cell exosomes, which were found to contain mRNA as well as miRNA [21]. Surprisingly, these molecules were functional and could transduce signals in recipient cells. Since then, exosomal RNAs have been implicated in the pathogenesis of a variety of important, chronic infections. For example, Eppstein-Barr virus-infected B-cells were shown to release exosomes containing viral miRNAs which could regulate gene expression in recipient cells [10]. *Toxoplasma gondii*-infected fibroblasts released exosomes containing a set of host mRNAs and miRNAs that was distinct from that of uninfected, serum-starved cells [12]. However, to date only two protozoan pathogens have been found to release RNA-containing extracellular vesicles directly. Thus, *Trichomonas vaginalis* exosomes were reported to contain RNA sequences, the biotype and function of which still remain to be determined [22], and *Trypanosoma cruzi* was shown to release extracellular microvesicles containing a variety of non-coding RNAs including tRNA-derived small RNAs, which have a suspected regulatory nature [16,23].

Based on the evidence that exosomes may serve as biologically important shuttle vectors for RNAs, in the present study, we sought to investigate the RNA content of leishmania exosomes. We indeed found that leishmania exosomes contained RNA cargo which they were capable of delivering to host cells *in vitro*. Using high throughput sequencing and bioinformatics analyses, we found that leishmania exosomes were enriched in small RNAs derived from largely non-coding RNAs. Notably, we discovered that these vesicles contained a relatively abundant and highly selective population of small RNAs derived from mature tRNAs. Furthermore, we found a number of novel transcripts, some of which were highly enriched in exosomes. Although exosomes released by both *L. donovani* and *L. braziliensis* had largely similar RNA content, *L. braziliensis* exosomes specifically contained transcripts derived from genes that also code for siRNAs.

Taken together, these findings show for the first time that leishmania exosomes are highly enriched in small non-coding RNAs, particularly tRNA-derived small RNAs with potential regulatory functions. This suggests that these RNAs may have functions in intercellular communication. These findings hint at a previously unrecognized potential mechanism of leishmania pathogenesis, mediated through the exosomal delivery of small, principally non-coding RNAs to mammalian host cells.

## Results

### *L. donovani* and *L. braziliensis* exosomes contain short RNA sequences; and intact vesicles protect their RNA cargo from degradation

We have previously reported that leishmania use an exosome-based secretion mechanism in order to export proteins with potential virulence properties [6,7]. Based on a number of studies in mammalian systems demonstrating the presence of RNA in exosomes, we were encouraged to expand on our findings and examine the RNA content of leishmania exosomes. We performed all experiments for this study with exosomes purified from supernatants of *L. donovani* or *L. braziliensis* cultured *in vitro* under infection-like stressors (acidic pH and elevated temperature for 24 h, see Methods), which induce the cells to transform into amastigotes. We had previously observed that these “early” axenic amastigotes release increased quantities of exosomes enriched in specific virulence factors [7]. Moreover, while undergoing transformation into amastigotes, leishmania modulate critical macrophage processes to allow for establishment of chronic infection. Combined with the fact that amastigotes are literally the only life cycle stage found *in vivo* in the mammalian host once infection is established, we felt that exosomes purified from this life cycle stage were the most relevant to examine.

**Figure 1:**
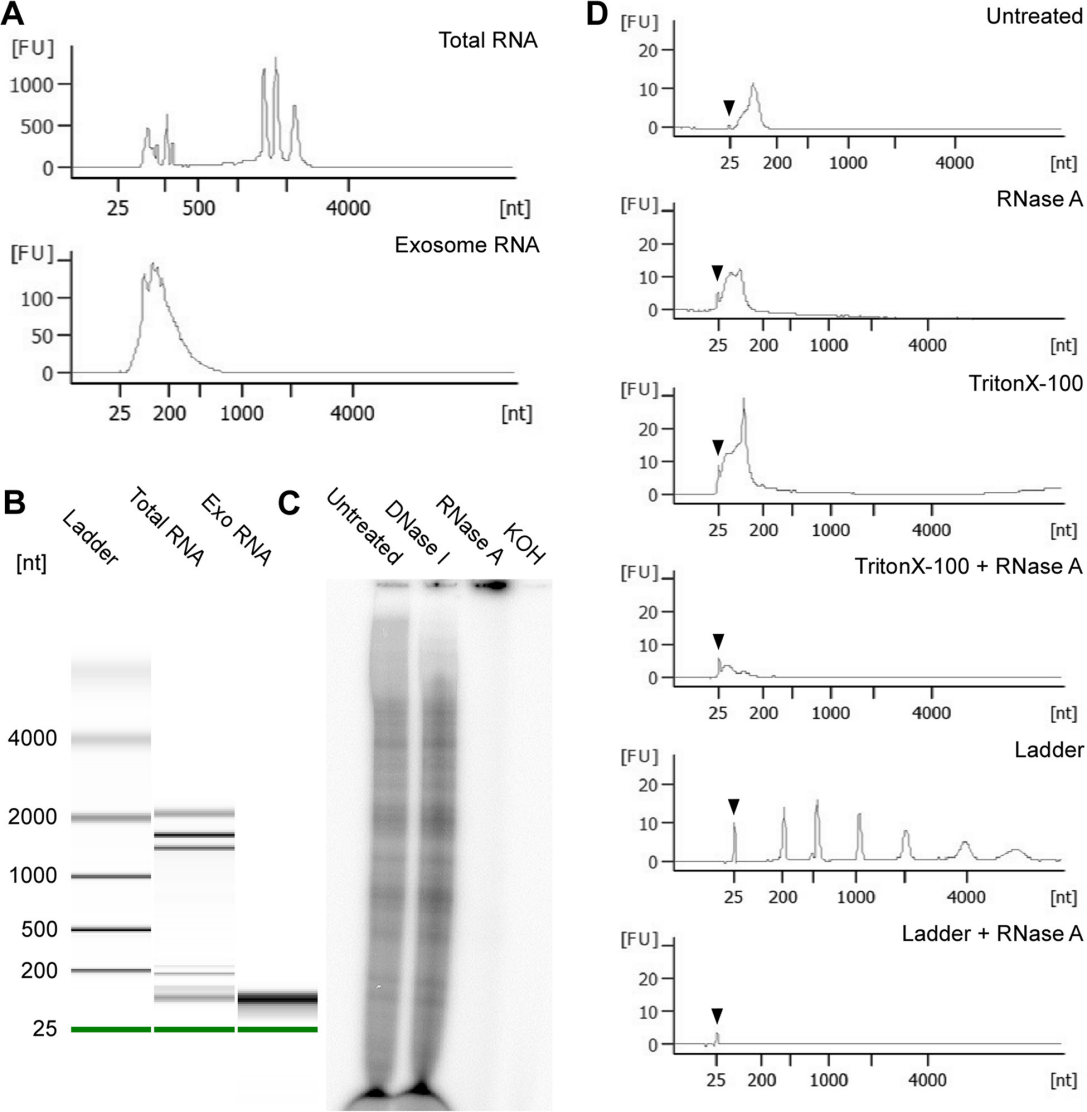
***L. donovani*** **exosomes contain RNA cargo.** Exosomes were purified from *L. donovani* axenic amastigote culture supernatant as described in the Methods. RNA was extracted from exosomes or whole cells by phenol-chloroform extraction and then analyzed. **A**. Agilent Bioanalyzer RNA length profiles of exosome RNA alongside total RNA (~100 ng RNA were loaded for each), **B**. Gel-like image from Agilent Bioanalyzer measurement, **C**. Purified exosome RNA (~250 ng/sample) was either left untreated or treated with DNase I, RNase A or KOH followed by radiolabelling with γ^32^P dATP and separation on a denaturing 15% polyacrylamide gel, **D**. RNA inside exosomes is resistant to degradation. Prior to RNA extraction, intact exosomes (purified from 400 mL culture supernatant) were either left untreated, or treated with RNase A or TritonX-100 or both. As a control for RNase A activity, 1 μL of the Agilent pico ladder was treated with the same concentration of RNase A. Samples were then subjected to RNA extraction and run on the Agilent Bioanalyzer. Arrowhead indicates internal 25 nt marker. nt, nucleotides. All images are representative of at least 3 independent experiments.

Exosomes were purified from supernatants of early axenic amastigotes and subjected to RNA extraction with phenol/chloroform. Results depicted in Figure 1A and B show that *L. donovani* axenic amastigote exosomes contained significant amounts of RNA that were detectable with the Agilent Bioanalyzer. Quantification with nanodrop revealed an average yield of 12.5 ng of RNA per μg of exosomal protein (data not shown). Notably, the length profile of exosomal RNA was distinct from that of *L. donovani* total RNA, with the bulk of exosomal sequences being short (25–250 nt). Furthermore, we did not detect full length ribosomal RNA (rRNA) peaks in exosome RNA profiles. In contrast, these full length rRNA peaks were prominent in the total RNA profiles. To confirm that the purified nucleic acid was in fact RNA, we incubated exosome RNA with DNase, RNase or KOH. As can be seen in Figure 1C, exosomal RNA was resistant to treatment with DNase, but was completely degraded upon exposure to either RNase or KOH.

To exclude the possibility that RNA was merely co-purified during exosome isolation but was not directly associated with or internal to the vesicles, we treated intact exosomes with RNase in the presence or absence of membrane-permeabilizing detergent. The results in Figure 1D show that when treated with RNase alone, exosome RNA remained intact. In contrast, when exosomes were treated with RNase and TritonX-100 simultaneously, the RNA signal was greatly diminished. These findings suggested that the RNA was confined within the exosomal membrane and thereby protected from degradation. The fact that we still saw a small residual signal after detergent and RNase treatment could indicate that a fraction of the RNA was bound to RNA-binding proteins and was thereby protected.

To investigate whether the release of RNA within exosomes is conserved between leishmania species, we purified and analyzed RNA from exosomes released by *L. braziliensis* early axenic amastigotes using the same procedures as described for *L. donovani*. This analysis showed that *L. braziliensis* exosomes also contain RNA, with similar characteristics to that of *L. donovani* exosome RNA (Additional file 1: Figure S1A and S1B). Taken together, these data represent the first description of RNA released by leishmania within exosomes.

### Leishmania exosomes deliver RNA cargo to human macrophages

**Figure 2:**
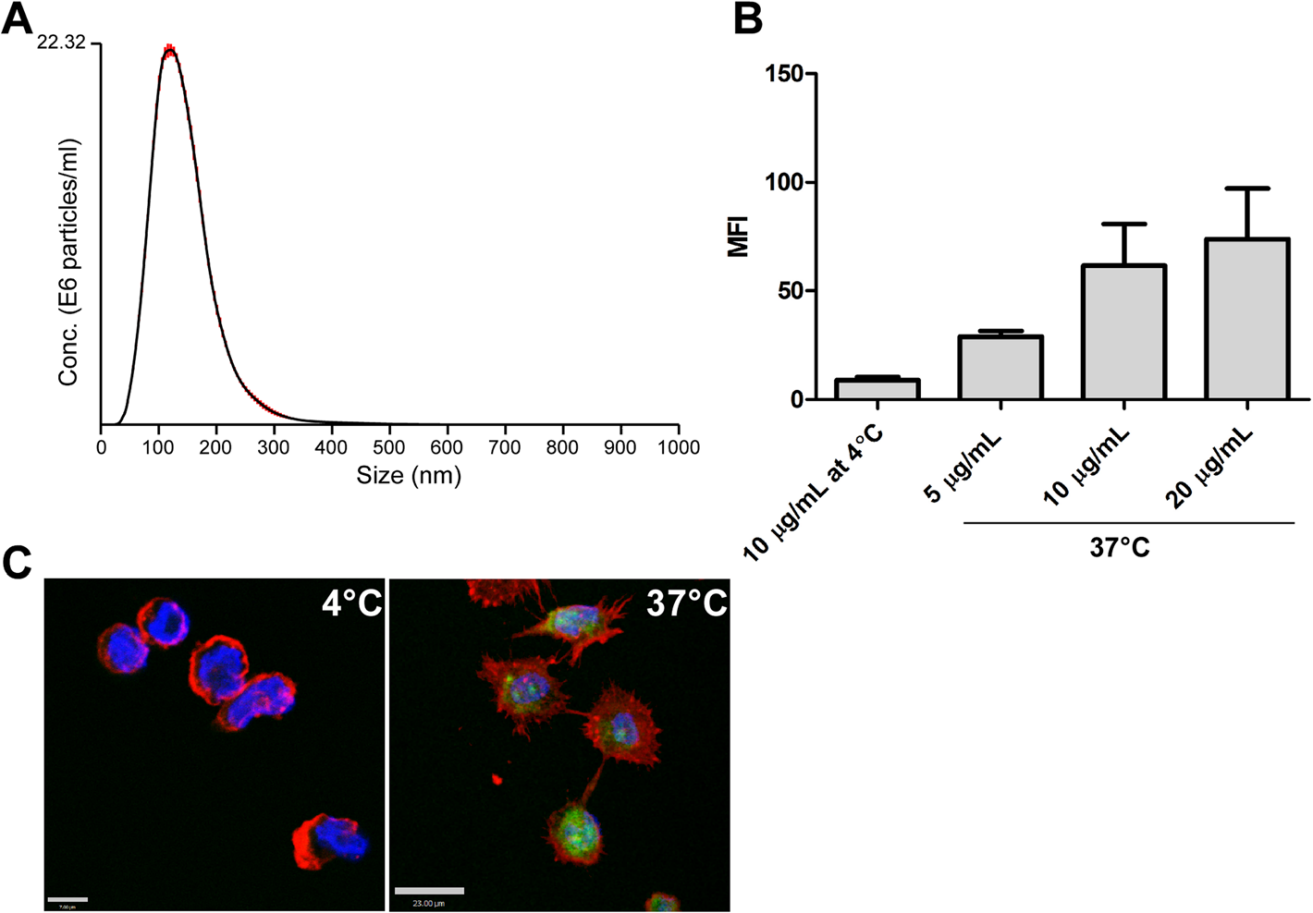
**Exosomal RNA cargo is delivered to macrophages.** Exosomes were purified from 400–800 mL supernatant of *L. donovani* axenic amastigotes, protein concentration was determined by Micro BCA, and exosomes were stained with a green fluorescent RNA-specific dye. PMA-differentiated THP-1 cells were incubated for 2 hours with labelled exosomes at either 37°C or 4°C to inhibit phagocytosis. **A**. Nanosight size profile of purified exosomes, **B**. Cells were treated with different concentrations of labelled exosomes as indicated and analysed by flow cytometry. Histograms were drawn, median fluorescence intensity (MFI) of cells was recorded, and the mean of 3 independent experiments was calculated (error bars represent standard error of the mean, SEM). **C**. Confocal microscopy of cells incubated with 10 μg/mL exosomes (green) at 4°C (left) or 37°C (right). Cells were stained with phalloidin-Alexa 594 to detect actin (red) and DAPI to detect nuclei (blue). Confocal microscopy was done with a Leica DMIRE2 inverted microscope equipped with a SP2 AOBS laser scanning head. Images were taken with a 63× magnification objective. Images are representative of 3 independent experiments.

In our previous studies, we observed that leishmania exosomes were released into infected macrophages and were taken up by uninfected bystander cells, and that exosomal proteins were delivered to host macrophage cytoplasm [7]. In order to investigate the potential delivery of exosomal RNA cargo to host macrophages, we labelled exosomes purified from the supernatant of *L. donovani* early amastigotes with an RNA specific fluorescent dye. Size and homogeneity of exosomes was assessed by Nanosight analysis (see Figure 2A) and the median size was determined to be 120 nm. Fluorescence of labeled exosomes was confirmed by microscopy (Additional file 2: Figure S2). PMA-differentiated THP-1 cells were incubated for 2 hours with fluorescently labelled exosomes and uptake was assessed by flow cytometry and confocal microscopy. As shown in Figure 2B, we observed a dose-dependent increase in fluorescence of cells, suggesting that macrophages readily take up exosomes and their RNA cargo. In contrast, control cells incubated at 4°C to inhibit phagocytosis showed only background fluorescence. To exclude the possibility that exosomes were just bound to the macrophage membrane but not internalized after incubation, we examined exosome-treated cells by confocal microscopy. Figure 2C shows that the fluorescence was localized to the cytoplasm of the macrophages and not to the membrane, indicating that the exosomes containing RNAs were indeed taken up by the cells. These results confirm that leishmania exosomes and their RNA cargo can be internalized by host cells and can access their cytoplasm.

### Characterization of leishmania exosome RNA cargo: Exosomes are enriched in small non-coding RNAs derived from tRNAs and rRNAs

**Figure 3:**
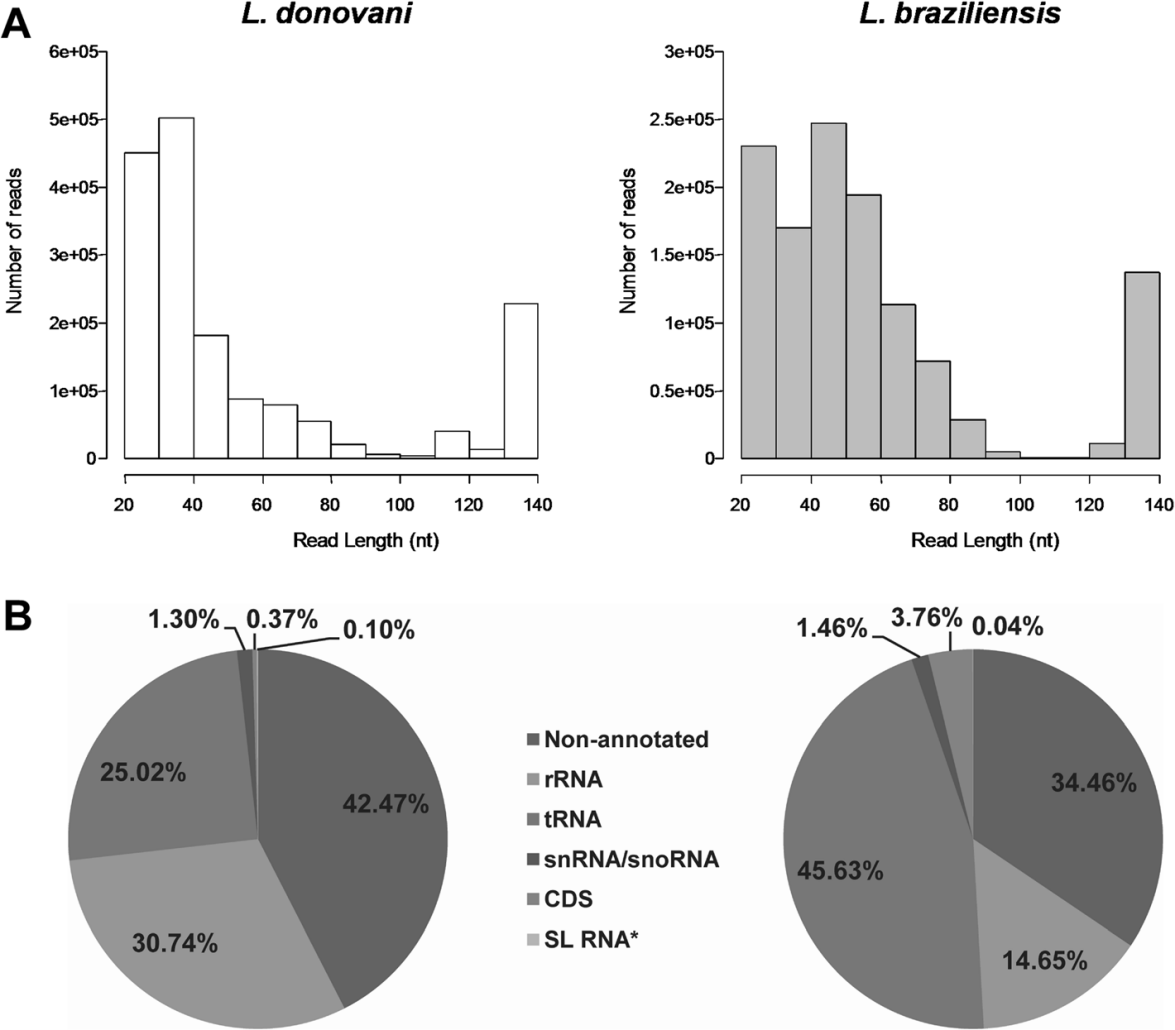
**Sequencing of leishmania exosomal RNA reveals conserved RNA cargo composed mainly of sequences derived from non-coding RNA.** Exosome RNA from *L. donovani and L. braziliensis* was purified and processed for high-throughput sequencing as described in Methods. **A**. Sequence length distribution of reads obtained from sequencing *L. donovani* and *L. braziliensis* exosome libraries, **B**. Categorization of reads according to their alignment with genomic features annotated in the *L. donovani* and *L. braziliensis* reference genomes. CDS, coding sequence; *SL RNA, spliced leader RNA. Numbers for reads mapping to SL RNA genes were obtained from alignment with the *L. major* reference genome, as these genes have currently only been annotated in this genome.

In order to assess the global transcriptome present in leishmania exosomes, we constructed complementary DNA libraries for high-throughput sequencing. We chose to compare RNA purified from exosomes released by early axenic amastigotes of *L. donovani* and *L. braziliensis* for three reasons: a) these two organisms cause distinct disease manifestations and hence can be expected to differ in their mechanisms of pathogenesis; b) they are spread through different vectors: *L. donovani* is transmitted by sandflies of the genus *Phlebotomus* in the Old World, whereas *L. braziliensis* is transmitted by *Lutzomyia* in the New World; and c) *L. braziliensis* was found to have a functional RNA interference pathway, which seems to be absent in *L. donovani* [24]. We hypothesized, therefore, that these two organisms could differ in their composition of exosomal RNA and chose to examine this directly. We used a strategy for library construction that was optimized for sequencing of small RNAs, as we had observed by gel electrophoresis that the exosomal RNA sequences were mainly short (Figure 1 and Additional file 1: Figure S1). We also incorporated a series of enzymatic treatments including dephosphorylation with calf intestinal alkaline phosphatase (CIP), 5′ cap removal with tobacco acid phosphatase (TAP) and 5′ re-phosphorylation with polynucleotide kinase (PNK) into the library construction procedure in order to pick up all sequences present in the exosomal transcriptome regardless of their 5′ modification (see Methods). Sequencing of the libraries by paired end 150 bp MiSeq Illumina sequencing resulted in ~1.4 million paired reads for *L. donovani* and ~1.1 million paired reads for *L. braziliensis* (Table 1). After adapter trimming and adjustment of the orientation of all reads to correspond to that of the original RNA sequence, reads were collapsed into unique reads prior to further analysis. As shown in the histograms in Figure 3A, read length distributions of reads were clearly skewed towards shorter reads with the mean read length being 55 nt for *L. donovani* and 57 nt for *L. braziliensis* (medians 37 nt and 49 nt, respectively).

**Table 1:** **Sequencing statistics**

	***L. donovani*** **library**	***L. braziliensis*** **library**
Total paired reads	1435277	1062571
Collapsed/unique reads	688524	538034
Unique and single copy	574049	421086

Numbers of reads for *L. donovani* and *L. braziliensis* exosome RNA libraries as obtained by high-throughput sequencing. Reads were combined into unique reads by collapsing all identical reads to one read for downstream analysis. This also revealed the number of reads that were present in the dataset as a single copy.

To get a general overview about what types of RNA transcripts were represented in our libraries, we aligned the reads of the *L. donovani* and the *L. braziliensis* libraries with reference genomes, respectively LdBPK (*Leishmania donovani* strain BPK282A1) and LbrM (*Leishmania braziliensis MHOM/BR/75/M2904*) using Bowtie 2 and the very-sensitive-local option which sets the seed length to 20 nucleotides, allowing for only one mismatch within the seed alignment (see Methods). We were able to align 58.61% of reads from the *L. donovani* library with the LdBPK reference genome and 22.87% of reads from the *L. braziliensis* library with the LbrM reference genome (see Additional file 3: Table S1A and S1B for the full datasets). The comparatively low alignment rate especially in case of the *L. braziliensis* library is likely a result of incomplete assembly of the reference genome or the fact that we used a different *L. braziliensis* strain (a clinical isolate from the Peruvian Amazon region) than the strain used to generate the reference genome. This is supported by the fact that we were able to align significantly more reads (52.88%) from the *L. braziliensis* library with the *L. major* reference genome (*Leishmania major* MHOM/IL/81/Friedlin, LmjF, see Additional file 3: Table S1B). Other possible causes for low alignment rates could be misinterpretation of modified nucleosides by the sequencer or RNA editing of sequences prior to packaging into exosomes, making it difficult to compare our transcriptomic data with the available reference genomes derived from DNA sequencing. RNA editing is a well-described process in leishmania and other trypanosomatids (e.g. [25,26]).

In order to ensure that our libraries were not contaminated with unrelated nucleic acids, we performed a BLAST search of all reads that failed to align with either the LdBPK, the LbrM or the LmjF reference genomes, against the NCBI nucleotide collection database (NCBI-NT). The results of this analysis showed that 28.1% of reads from the *L. donovani* exosome library and 36.3% of reads from the *L. braziliensis* exosome library aligned to sequences in the NCBI-NT database (see Additional file 4: Table S2A and S2B). Of these, 4.93% of *L. donovani* and 4.17% of *L. braziliensis* aligned with other leishmania genomes. The rest aligned with a promiscuous group of >6000 different plant, fungi, helminth and bacteria species, several of which were plant pathogens or soil inhabitants. Based on the observation that there was no enrichment of any particular species and that overall, the majority of reads from both libraries aligned with leishmania genomes (in total 63.54% of reads of the *L. donovani* library and 57.05% of the *L. braziliensis* library, see summary of alignment statistics in Additional file 5: Table S3), we concluded that we did not have a contamination issue that would impugn our data. We think that many, if not all, of the reads mapping to bacteria or helminth genomes are likely false positive hits. Thus, even though our alignment rates were somewhat lower than we might have expected, we think that our datasets are valid and large enough to draw meaningful conclusions about the exosomal RNA content.

When categorizing reads into RNA biotypes based on reference genome annotations, we saw that for both libraries, the majority of reads were aligning with rRNA and tRNA genes, in the sense orientation (Figure 3B). In addition, a large number of reads mapped to non-annotated (intergenic) regions of the reference genomes (42.47% for *L. donovani* and 34.46% for *L. braziliensis*), which could potentially be novel transcripts. Interestingly, we only saw less than 4% of reads mapping to protein coding genes (CDS) or spliced leader (SL) RNA genes. These results indicated that the majority of sequences present in the leishmania exosome transcriptome are derived from non-coding RNAs and intergenic regions, whereas sequences derived from mRNAs are underrepresented.

When working with the LdBPK and LbrM reference genomes, we had to take into account that both are limited in their annotations. Hence, it was not surprising that we found a large number of reads in our libraries mapping to intergenic regions. Whereas the annotations of CDS are thought to be comprehensive in these genomes, the assignments of SL RNAs as well as structural non-coding RNAs such as rRNAs, tRNAs, snRNAs and snoRNAs are clearly lacking in completeness. Consequently, it has to be considered that the large number of reads mapping to intergenic regions may not necessarily all be novel transcripts, but could also have resulted from incomplete annotation of non-coding RNA types in these regions. Keeping this in mind and still trying to dissect what types of RNA sequences are highly represented in exosomes, we decided to inspect in greater detail the alignment of the most abundant exosomal sequences manually using the Artemis genome browser software [27]. For this purpose, reads were clustered into unique regions of alignment and the regions were ranked by abundance (number of reads found per region). Considering that *L. major* is the species with the best assembled genome to date and presents the most complete annotation of non-coding RNAs, we also performed alignments of *L. major* annotated non-coding RNAs with the LdBPK and LbrM reference genomes, in order to identify non-annotated, non-coding RNA loci in our target genomes. The results of the screening using Artemis showed that the top 20 most abundant reads from both libraries mapped to three RNA classes in the sense orientation: rRNA, tRNA and snRNA (Table 2).

**Table 2:** **Top 20 most abundant clusters of transcripts present in leishmania exosomes**

***L. donovani***						***L. braziliensis***					
**Chr**	**Coordinates of genomic locus**		**Annotation**	**No. of reads**	**RNA biotype**	**Chr**	**Coordinates of genomic locus**		**Annotation**	**No. of reads**	**RNA biotype**
**27**	1014367	1019133	LdBPK_27rRNA3	344191	28S rRNA	**6**	334041	334897	LmjF.27.rRNA.31	58906	28S rRNA
			LdBPK_27rRNA4						LmjF.27.rRNA.34		
			LmjF.27.rRNA.13								
			LmjF.27.rRNA.22								
			LmjF.27.rRNA.29								
			LmjF.27.rRNA.31								
			LmjF.27.rRNA.33								
			LmjF.27.rRNA.42								
**27**	1019947	1021495	LdBPK_27rRNA6	132730	18S rRNA	**00**	463646	464099	LbrM.27.rRNA1	11166	18S rRNA
**15**	312758	313248	LmjF.15.TRNAASP.01	65737	tRNA-Asp	**15**	324587	325394	LbrM.15.tRNA1	18229	tRNA-Asp
			LmjF.15.TRNAGLU.01		tRNA-Glu				LbrM.15.tRNA2		tRNA-Glu
			LmjF.09.5SrRNA.02		5S rRNA				LbrM.15.rRNA1		5S rRNA
			LmjF.05.5SrRNA.01								
			LmjF.15.5SrRNA.01								
**24**	715730	715801	LdBPK_24tRNA5	43207	tRNA-Asp	**24**	659346	659417	LbrM.24.tRNA5	30583	tRNA-Asp
**17**	328838	328909	LdBPK_17tRNA1	42601	tRNA-Asp	**17**	296223	296604	LbrM.17.tRNA1	30523	tRNA-Asp
									LbrM.17.tRNA2		tRNA-Ser
									LbrM.17.tRNA3		tRNA-Ala
**24**	658796	658976	LdBPK_24tRNA2	35611	tRNA-Gln	**24**	600448	600615	LbrM.24.tRNA2	8749	tRNA-Gln
**09**	429809	430355	LdBPK_09tRNA6	29246	tRNA-Glu	**09**	395278	395779	LbrM.09.tRNA3	4467	tRNA-Val
			LmjF.09.TRNAARG.01		tRNA-Arg				LbrM.09.tRNA4		tRNA-His
			LmjF.09.TRNAVAL.02		tRNA-Val				LbrM.09.rRNA1		5S rRNA
			LmjF.05.5SrRNA.01		5S rRNA				LmjF.05.5SrRNA.01		
			LmjF.11.5SrRNA.03						LmjF.11.5SRRNA.03		
			LmjF.21.5SrRNA.01						LmjF.21.5SrRNA.02		
						**09**	403494	403565	LbrM.09.tRNA5	5226	tRNA-Glu
**31**	495812	496115	LdBPK_31tRNA3	18528	tRNA-Glu	**31**	582437	582738	LbrM.31.tRNA2	4719	tRNA-Gly
									LbrM.31.tRNA3		tRNA-Glu
**27**	1019543	1019804	LdBPK_27rRNA5	18473	5.8S rRNA						
**11**	156707	157038	LmjF.11.TRNAALA.01	15493	tRNA-Ala	**11**	63421	63678	LmjF.33.TRNAALA.01	3041	tRNA-Ala
			LmjF.36.TRNALEU.01		tRNA-Leu				LmjF.11.TRNALEU.02		tRNA-Leu
**27**	1014054	1014340	LmjF.27.rRNA.47	14260	28S rRNA						
			LmjF.27.rRNA.48								
**23**	230438	230509	LdBPK_23tRNA9	13814	tRNA-Gly	**23**	216842	216916	LbrM.23.tRNA9	3679	tRNA-Gly
**36**	1630332	1630403	LdBPK_36tRNA2	11529	tRNA-Gln						
**05**	360707	361335	LdBPK_05snRNA1	9971	snRNA	**05**	349991	350587	LbrM.05.rRNA1-1	6306	5S rRNA
									LbrM.05.ncRNA1-1		ncRNA
**33**	104560	104930	LdBPK_33tRNA1	9352	tRNA-Ala	**33**	105787	105859	LbrM.33.tRNA1	5730	tRNA-Arg
			LdBPK_33tRNA2		tRNA-Arg						
			LdBPK_33tRNA3								
**23**	229645	229857	LdBPK_23tRNA5	8487	tRNA-Leu	**35**	2472707	2472788	LbrM.35.tRNA4	2592	tRNA-Leu
			LdBPK_23tRNA6		tRNA-Thr						
**16**	445957	446028	LdBPK_161tRNA1	7916	tRNA-Gln	**16**	442089	442160	LbrM.16.tRNA1	8695	tRNA-Gln
**23**	230585	230656	LdBPK_23tRNA10	7804	tRNA-Trp	**23**	216992	217063	LbrM.23.tRNA10	3657	tRNA-Trp
						**21**	430678	430798	LbrM.21.rRNA1	3273	5S rRNA

Reads were clustered into genomic loci based on Bowtie 2 alignments with reference genomes (as described in Methods) to identify the RNA biotypes that were most abundant in exosomes. The details of the top 20 clusters with the highest numbers of reads falling into them are listed. Clusters of reads in the *L. donovani* library are listed in descending order of abundance, with the homologous cluster of reads in the *L. braziliensis* library given in the same row. Chr = chromosome number, annotation = annotation in reference genomes (LdBPK = *L. donovani*, LbrM = *L. braziliensis* or LmjF = *L. major*) followed by the gene name, No. of reads = number of reads from the respective library falling into this cluster, RNA biotype = type(s) of RNA that is annotated in the reference genomes in this region.

The high abundance of reads mapping to rRNA genes observed in both libraries is in compliance with other recent reports on RNA types found in exosomes. Upon closer inspection we saw that the majority of reads mapping to rRNA genes were shorter fragments (median length 39 nt for the *L. donovani* library and 52 nt for *L. braziliensis*, see Additional file 6: Figure S3). We then looked for enrichment of specific rRNA genes within our pool and saw that the majority of reads from both libraries mapped to 28S and 18S rRNA genes (>90%, Additional file 7: Table S4). Furthermore, we investigated the position of alignment of reads within the various rRNA genes, and found that for both libraries, reads aligned along the entire length of these genes (Additional file 7: Table S4). It was of particular interest to find a large number of reads mapping to tRNAs in both libraries, as tRNA-derived small RNAs have recently been discovered in *T. cruzi* [28,29], and these novel small RNAs are thought to participate in regulation of gene expression [23,30] (see below for a more detailed analysis of tRNA-derived small RNAs).

Notably, to our surprise, the overlap of the RNA profiles for *L. donovani* and *L. braziliensis* was striking. Thus, these parallel and independent RNA-seq replicates provide direct evidence for the reproducibility of our data.

### Exosomes carry putative novel transcripts

**Figure 4:**
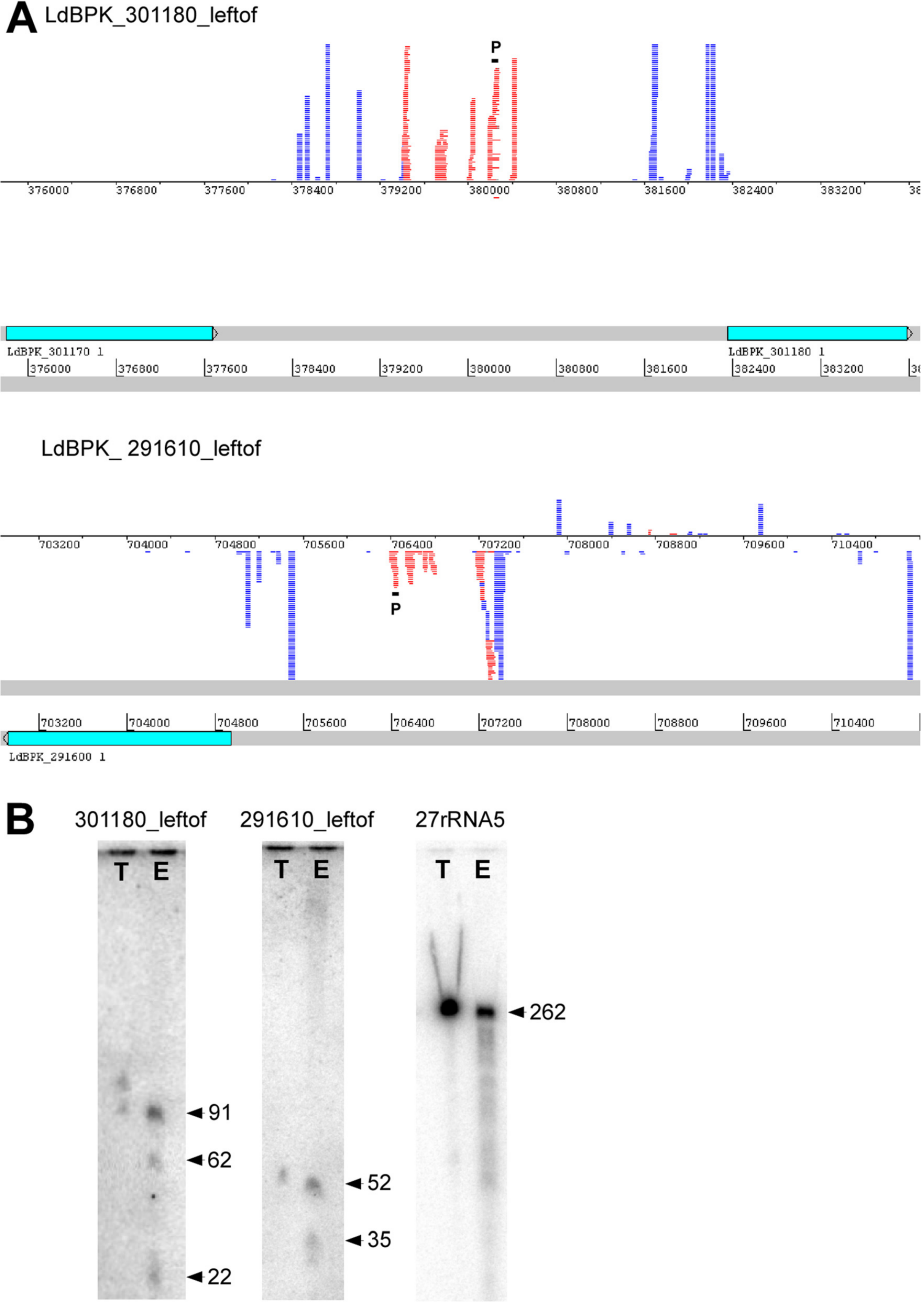
**Novel transcripts are found in leishmania exosomes. A**. Artemis genome browser alignments of the *L. donovani* exosome library and a *L. donovani* spliced leader sequence library (P. Myler, unpublished) with the *L. donovani* reference genome. Shown are two regions with reads mapping to them. Top: intergenic region on chromosome 30 (LdBPK_301180_leftof), bottom: intergenic region on chromosome 29 (LdBPK_291610_leftof). Light blue boxes on grey tracks are annotated genes. In dark blue are the reads from the spliced leader library, in red the reads from the exosome libraries. “P” designates the regions that were used for designing probes for Northern blotting. **B**. Northern blots with probes designed for novel transcripts found in exosomes, corresponding to the genomic regions shown in panel **A** (301180_leftof and 291610_leftof), plus an additional probe against a 5.8S rRNA (27rRNA5). *L. donovani* total (T) and exosome (E) RNA were probed on the same membrane. Equal amounts of RNA (3 μg) were loaded in each lane. Sizes of bands on membranes as indicated are in nucleotides (nt) and were calculated based on 262 nt, 150 nt and 21 nt size markers.

To make sure we did not miss any important information amongst the group of less abundant reads, we randomly selected a number of less abundant reads from both libraries and inspected their alignment with the reference genomes manually. Interestingly, we discovered several reads mapping to intergenic regions at different genomic loci (Table 3). These intergenic regions were neither annotated at those loci in any of the sequenced leishmania or trypanosome genomes, nor did they share homology to any known trypanosomatid gene (as assessed by performing BLAST searches on TriTrypDB and NCBI). These findings suggested that the sequences mapping to these regions corresponded to *bona fide* novel transcripts. Notably, we found homologous novel transcripts in both libraries, providing evidence that they are both conserved between species as well as packaged into exosomes. When overlaying our sequencing data from the *L. donovani* exosome RNA library with a recently sequenced *L. donovani* spliced leader (SL) RNA library (P. Myler, unpublished data), we observed that the genomic loci giving rise to our identified novel transcripts had SL sites in the 5′ region upstream of them (see Figure 4 for 2 examples). This indicates that they might be processed by *trans*-splicing and are hence likely to be functional mature transcripts rather than promiscuous transcriptional by-products.

**Table 3:** **Intergenic regions coding for putative novel transcripts in exosomes**

***L. donovani***					***L. braziliensis***				
**Name**	**Coordinates**		**No. of reads**	**No. of ORF**	**Name**	**Coordinates**		**No. of reads**	**No. of ORF**
**LdBPK_301180_leftof**	379397	380435	363	7	**LbrM.30.1240_leftof**	394563	397304	317	13
**LdBPK_291610_leftof**	706385	707360	181	9	**LbrM.29.1600_leftof**	663702	664543	103	7
**LdBPK_360420_leftof**	109068	109733	139	3	**LbrM.35.0480_leftof**	132138	133299	185	5
**LdBPK_363000_leftof**	1183324	1183522	132	1	**LbrM.35.3080_leftof**	1176690	1176833	36	1
**LdBPK_362290_leftof**	872006	872406	89	5	**LbrM.35.2400_leftof**	892711	895894	29	28
**LdBPK_313190_leftof**	1452630	1452719	83	1	**LbrM.31.3490_leftof**	1508257	1510948	0	N.A.
**LdBPK_040550_leftof**	225336	230737	80	8	**LbrM.04.0610_leftof**	229925	233378	111	25
**LdBPK_131560_leftof**	555895	556192	75	2	**LbrM.13.1200_leftof**	433509	433481	0	N.A.
**LdBPK_364270_leftof**	1570740	1570991	57	1	**LbrM.35.4310_leftof**	1563654	1563940	29	2
**LdBPK_366120_leftof**	2270195	2272110	49	13	**LbrM.35.6160_leftof**	2242345	2244214	153	14
**LdBPK_330560_leftof**	173136	173362	40	0	**LbrM.33.0550_leftof**	184438	184751	19	1
**LdBPK_366590_leftof**	1903364	1906720	0	N.A.	**LbrM.35.6630_leftof**	2438694	2438825	155	0

List of 12 intergenic regions identified with numbers of reads mapping to them listed by descending order of abundance in the *L. donovani* library, with the homologous genomic region in the *L. braziliensis* library given in the same row. Names are derived from the annotated genes adjacent to the intergenic region plus the designation “_leftof”, indicating that the intergenic region is on the left side of the annotated gene on the same strand, regardless of transcriptional direction. There is 75% overlap of intergenic regions coding for novel transcripts in the *L. donovani* and *L. braziliensis* libraries. ORF, open reading frame. N.A., not applicable.

We also searched for open reading frames (ORF) within the sequences of the novel transcripts to see whether they have the potential to code for a protein or peptide and found potential ORFs for the majority of them (Additional file 8: Table S5). However, when we translated the ORFs and looked for homologies to known proteins in the NCBI database using Blastp, we did not obtain any hits.

Based on the hypothesis that these novel transcripts could have a role in regulation of gene expression in either the mammalian or insect host or both, we performed Bowtie2 alignments to the human and the vector (Lutzomyia and Phlebotomous) genomes to search for potential targets in these genomes, looking for complementarity. We obtained 60 hits for all of the 1288 reads representing novel transcripts in the *L. donovani* library when searching against the human genome and 15 hits when searching against the Phlebotomus genome (Additional file 9: Table S6A). For the 1137 reads that comprise the novel transcripts in the *L. braziliensis* library, 25 hits were observed when searching against the human genome and 6 hits when searching against the Lutzomyia genome (Additional file 9: Table S6B). However, nearly all of these hits were determined to be in non-annotated regions of the respective genomes, implying – at least for the human genome - that there are no genes that could be regulated by the novel transcripts, based on the parameters applied in our analyses (perfect complementarity). Of note, the annotation of the vector genomes is a very recent effort and far from complete. It is quite possible, therefore, that there are as yet non-annotated protein coding genes in the genomic regions where the novel exosomal transcripts aligned, which would have been missed, resulting in false negative findings. Moreover, in most animals, regulatory RNAs such as miRNAs have incomplete homology with their target sequences [31] and, therefore, our predictions based on perfect complementarity may have missed some potential targets in the host genomes, again leading to false negative results. Unfortunately, as the tools to predict RNA-RNA interactions at the level of potential regulatory RNA-mRNA target pairs are fairly limited (generating a massive amount of ambiguous results when analysing large datasets), we were unable to carry out a comprehensive host mRNA target prediction with the novel transcripts that was informative. We also performed an alignment search of the novel transcripts against the databases of human and mouse miRNAs (mirbase.org), but failed to detect homologous sequences. All of the novel transcripts we identified were present in the sense orientation of transcription in leishmania, which implies that they are unlikely to be present in exosomes as double strands, which is a characteristic of canonical siRNAs and miRNAs.

To validate the presence of the identified novel transcripts in exosomes and compare their expression in exosomes with total leishmania RNA, we designed probes for Northern blotting. We selected the two most abundant novel transcripts identified in the *L. donovani* exosome library, one of which was positioned in between the genes 1170 and 1180 on chromosome 30 (LdBPK_301180_leftof) and the other in between the genes 1600 and 1610 on chromosome 29 (LdBPK_291610_leftof) for probe design. The regions that are complementary to the probes used are indicated in Figure 4A. We loaded equal amounts of *L. donovani* total and exosome RNA into a polyacrylamide gel, along with 21 nt and 150 nt size markers. The results from the Northern blots showed that both novel transcripts could be detected in total and exosome RNA (Figure 4B). Of interest, we detected bands of larger size that were present in both total and exosome RNA (which likely represent the primary transcript), but where the signal appeared to be stronger in the exosomal RNA lane. In addition, we detected bands of smaller size in the exosome RNA, that were completely absent in the total RNA. For comparison, we also incubated a blot with a probe for 5.8S rRNA (LdBPK_27rRNA5, Ref), which appeared to be much more abundant in total than in exosome RNA. These results confirm the presence of the novel transcripts identified by sequencing in exosomes and indicate that fragments of these transcripts with specific lengths are uniquely present in exosomes, consistent with selective packaging.

### *L. braziliensis* exosomes carry a low abundance of sequences derived from siRNA-coding regions

As discussed above, *L. braziliensis* can regulate gene expression through the RNAi pathway and produce small interfering RNAs (siRNAs) [32]. We were interested in exploring the possibility to find siRNAs as part of *L. braziliensis* exosomal RNA cargo. The main classes of *L. braziliensis* siRNAs are derived from the spliced leader-associated conserved sequence (SLACS) retroposon [33], the telomere-associated transposable element (TATE) [33], the *L. braziliensis*-specific telomere-associated sequence (TAS) [34] and the chromosomal internal repeats, 74-nucleotide long (CIR74) [32]. We generated a BLAST database from a FASTA file with 41 SLACS/TATEs extracted from TriTrypDB-5.0_LbraziliensisMHOMBR75M2904_ AnnotatedTranscripts.fasta comprising the nucleotide sequences of the SLACS and TATEs genetic elements and performed a BLAST search with our libraries against this database. In the *L. braziliensis* library, we found 4471 reads mapping to these elements (Additional file 10: Table S7). Interestingly, about 50% of these reads were both sense and antisense, suggesting that the sequences were present in exosomes as double-stranded RNAs. The lengths of reads were somewhat heterogeneous, ranging from 20 nt to 70 nt, whereas *L. braziliensis* mature siRNAs (*Lbr*AGO1-bound) are believed to be 20–25 nt in length [32]. For comparison, we also performed the same BLAST analysis with the *L. donovani* library, where we only found 353 reads mapping to the siRNA-coding genetic elements (Additional file 10: Table S7). The fact that we found some reads in the *L. donovani* library mapping to these elements could be due to settings used for the BLAST search that were not stringent enough (cut off 80% identity and 70% query coverage), possibly resulting in false positive alignments. Despite this, it is clear that there were >10 times more reads in the *L. braziliensis* library mapping to siRNA-coding genetic elements, indicating that our results are specific and providing evidence that *L. braziliensis* may export siRNAs or their precursors within exosomes. However, we cannot rule out the possibility that the sequences we found in exosomes may originate from regions of the SLACS/TATEs genes other than the ones giving rise to siRNAs.

### Leishmania exosomes contain an abundance of specific tRNA-derived fragments

**Figure 5:**
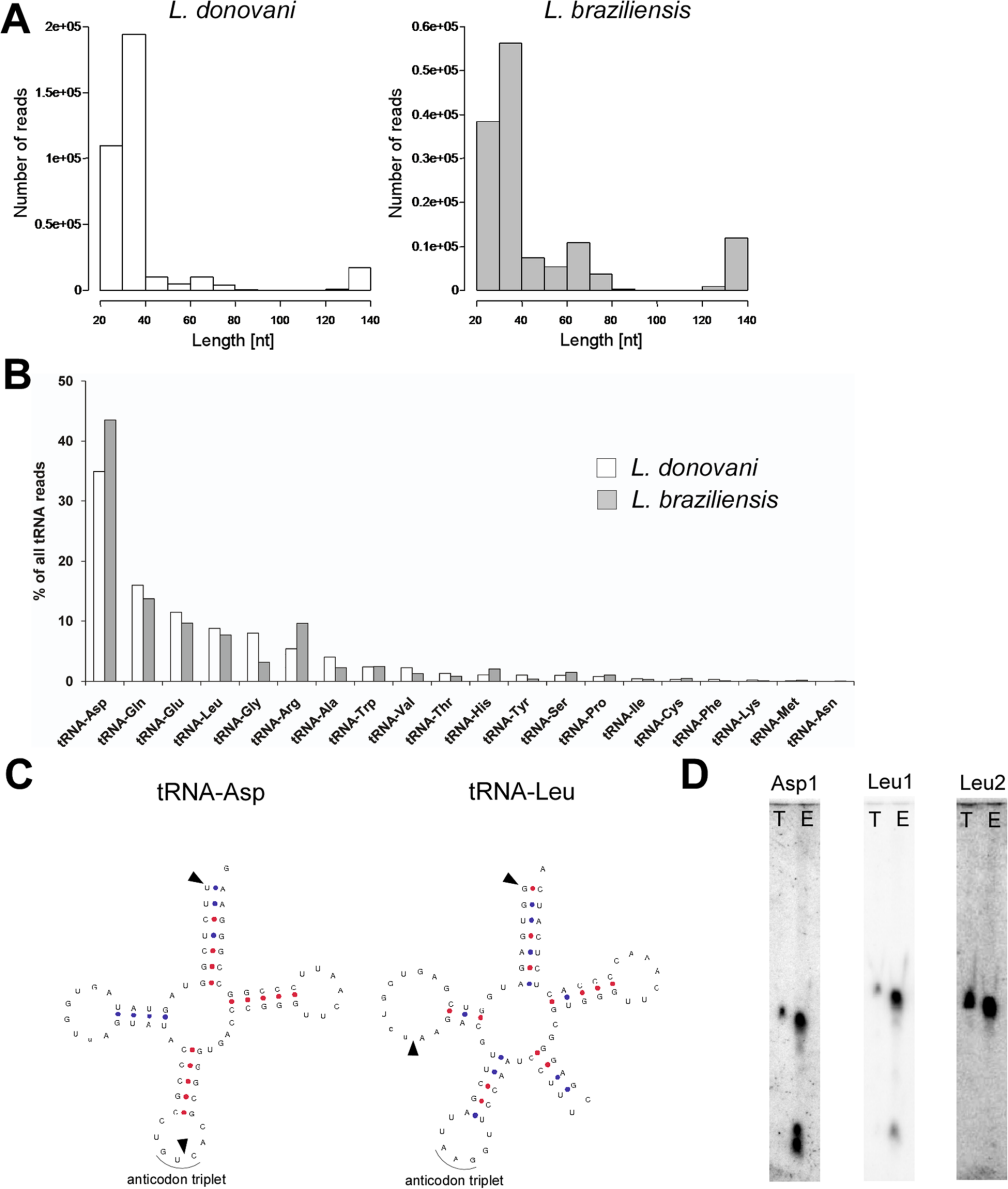
**tRNA-derived fragments are cargo of leishmania exosomes. A**. Length distributions of reads mapping to tRNAs in *L. donovani* and *L. braziliensis* exosome RNA sequencing libraries. **B**. Bar graph showing percentages of reads from *L. donovani* (white bars) and *L. braziliensis* (grey bars) mapping to the respective tRNA isoacceptors. **C**. tRNA secondary structures for leishmania tRNA-Asp and tRNA-Leu (downloaded from [38]). Arrowheads indicate major cleavage products as observed in the sequenced libraries. **D**. Northern blots with probes designed against tRNA-Asp (Asp1) and tRNA-Leu (Leu1 and Leu2). *L. donovani* total (T) and exosome (E) RNA were probed on the same membrane. Equal amounts of RNA (3 μg) were loaded in each lane. Asp1 and Leu1 probes were designed to specifically detect full length tRNA as well as t-RNA-derived small RNAs seen in sequencing libraries, whereas Leu2 was designed against the mid region (anticodon loop) of tRNA-Leu and hence only detects the full length tRNA.

Remarkably, we found a large number of reads in both the *L. donovani* and *L. braziliensis* exosome RNA libraries that mapped to tRNA genes. A few recent studies characterizing the RNA content of mammalian exosomes had reported the presence of tRNAs or their fragments in these vesicles. For example, reads mapping to tRNAs were found in sequencing libraries made with RNA from exosomes released from neuronal cells (13.5%) [35], immune cells (~7%) [36] and plasma exosomes (1.24%) [37]. Strikingly, in our datasets, 351,919 reads (36.4 %) and 135,149 reads (21.1%) from *L. donovani* and *L. braziliensis*, respectively, mapped to tRNA genes when aligned to the *Leishmania major* MHOM/IL/81/Friedlin (LmjF) reference genome (which has the best curation on tRNA annotation amongst leishmania species). These frequencies exceeded by some measure those reported for mammalian exosomes in the studies cited above. Close inspection of the genome alignments revealed that a high percentage of these sequences were covering only parts of the respective tRNA genes (Figure 5), consistent with the occurrence of tRNA-derived small RNAs (tsRNAs), which has recently been recognized as a specific process. In light of these findings we decided to characterize the reads mapping to tRNAs in more detail. In case of both libraries, the vast majority (99.8%) of reads were in the sense direction of transcription. Looking at their length profiles, we found the mean read length to be slightly different between the two libraries, 38 nt for *L. donovani* and 46 nt for *L. braziliensis*, however, the median read length was similar (33 nt and 34 nt, respectively) (Figure 5A). For both leishmania libraries, tsRNAs derived from tRNA-Asp, tRNA-Gln, tRNA-Glu and tRNA-Leu were most abundantly present (Figure 5B and Table 4). To make a case that these tsRNAs were specific cleavage products that are selectively packaged into exosomes and not just a by-product of tRNA turnover that is disposed by the cell, we calculated the Pearson’s correlation of the predicted cellular amino acid usage and the relative expression of our tsRNAs as assessed by sequencing. The results showed that there was no correlation (*r* = 0.163 for *L. donovani* and *r* = 0.114 for *L. braziliensis*), indicating that the tsRNAs were unlikely to be random degradation products. Strikingly, we observed the same rank order frequency of tRNA isotypes as origins of tsRNAs in both libraries (Figure 5B and Table 4), indicating that the formation of specific tsRNAs and their appearance as exosomal cargo is an evolutionary conserved phenomenon in leishmania.

**Table 4:** **Reads mapping to tRNAs**

	***L. donovani*** **library**			***L. braziliensis*** **library**		
**tRNA**	**% of total tRNA reads**	**Length [nt]** ^**A**^	**Position** ^**B**^	**% of total tRNA reads**	**Length [nt]** ^**A**^	**Position** ^**B**^
**Asp**	34.91	47	5′	43.51	58	5′
**Gln**	16.00	32	mid-5′	13.73	36	mid-5′
**Glu**	11.48	38	3′	9.65	46	5′
**Leu**	8.80	29	5′	7.66	31	5′
**Gly**	8.02	37	3′	3.11	43	5′
**Arg**	5.40	31	mid-5′	9.62	28	mid-5′
**Ala**	4.03	34	3′	2.21	34	3′
**Trp**	2.38	37	mid-3′	2.42	40	mid-3′
**Val**	2.24	36	5′	1.28	41	5′
**Thr**	1.33	31	3′	1.49	35	3′
**His**	1.08	39	mid-3′	1.99	42	mid-3′
**Tyr**	1.06	31	3′	0.38	28	3′
**Ser**	1.02	34	3′	0.82	39	mid-3′
**Pro**	0.80	31	3′	1.02	34	3′
**Ile**	0.44	25	3′	0.31	31	3′
**Cys**	0.34	25	mid	0.48	26	mid
**Phe**	0.32	36	3′	0.07	36	3′
**Lys**	0.24	36	3′	0.09	37	3′
**Met**	0.10	29	3′	0.15	33	3′
**Asn**	0.01	33	mid-5′	0.01	38	mid-3′

^A^Average read length.

^B^Most abundant read position (of all tRNA reads).

Distribution of reads from *L. donovani* and *L. braziliensis* libraries over different tRNA isoacceptors. These are sorted by descending abundance in the *L. donovani* library, with the equivalent reads from the *L. braziliensis* library in the same row. nt = nucleotide. N.A. = not applicable.

Next, we investigated whether the tsRNAs were derived from the 3′ or 5′ end of mature tRNAs, and found that the most abundant tsRNA^Asp^ and tsRNA^Gln^ were derived from the 5′end or mid-5′ end in both libraries (Table 4). Notably, this was not the case for all of the tsRNAs, as some appeared to be derived from the 3′ end, and two were not derived from the same end in the two different species (tsRNA^Glu^ and tsRNA^Gly^) (Table 4). Furthermore, we saw tsRNAs of different lengths, some of them corresponding to the length of tRNA-halves (~30 nt, e.g. tsRNA^Leu^), and others to the length of tRNA-derived RNA fragments (25 nt, e.g. tsRNA^Ile^) (Table 4).

Based on the hypothesis that leishmania tsRNAs may act as miRNAs or siRNAs in the mammalian or invertebrate host we performed additional Bowtie2 alignments with all reads mapping to leishmania tRNAs against the human and vector genomes looking for complementarity to find potential targets for these potential regulatory RNAs. This search yielded a very large number of hits (~20,000), the majority of which fell into non-annotated regions of the host genomes (Additional file 9: Table S6A and S6B). Analogous to our target prediction analyses with the novel transcripts described above, we were unable to perform a more comprehensive (based on more complex RNA-RNA interactions rather than simple complementarity alone) analysis of potential leishmania tsRNA-host target mRNA interactions due to the lack of appropriate tools for large datasets. We also performed a miRNA homology search against the human and mouse miRNA database, and found only one miRNA (miR-135b-5p) that shared 88% identity with one of the tsRNAs present in both libraries (tsRNA^Arg^) (Additional file 11: Table S8). These results indicate that leishmania tsRNAs are not highly similar to canonical mammalian miRNAs.

In order to validate the presence of the identified tsRNAs in exosomes and to compare their abundance in exosomes with total RNA, we performed Northern blotting with probes specific for tsRNA^Asp^ (Asp1) and tsRNA^Leu^ (Leu1). The probes were designed to be complementary to the 5′ end of each tRNA and hence recognize both full length tRNA and 5′ tsRNA. We also included a probe that was designed against the middle region (anticodon loop) of tRNA-Leu for comparison (Leu2). When hybridizing blots of RNA isolated from *L. donovani* total cells and versus exosomes with these probes, we detected a common band corresponding to the full length tRNA in total and exosome RNA in case of both the probes Asp1 and Leu1 (72 nt and 82 nt respectively) (Figure 5D). In addition, we detected two smaller bands in the exosome RNA lane of the blot probed with Asp1 which were absent in the total RNA lane. Similarly, we detected a smaller band in the exosome RNA lane of the blot probed with Leu1 which was absent in the total RNA lane. These results demonstrate that 5′ tsRNAs are produced from tRNA-Asp and tRNA-Leu and that these tsRNAs are highly enriched in exosomes. In the blot probed with Leu2 we only detected a band corresponding to the full length tRNA in both total and exosome RNA, confirming the absence of fragments that are derived from the anticodon loop of this tRNA. In summary, these findings are the first to show that leishmania produce tRNA-derived small RNAs and that tsRNAs are specifically enriched in exosomes.

## Discussion

### Leishmania exosomes contain specific RNA cargo

It has been firmly established that exosomes released by various mammalian cell types can serve as shuttle vehicles to deliver RNA molecules to recipient cells, thereby influencing gene expression. However, to date no protozoan pathogen has been shown to release *bona fide* exosomes containing RNAs with gene regulatory or other sequence-specific properties. Leishmania have recently been shown to secrete exosomes that contain a plethora of protein virulence factors capable of affecting the phenotype of host mononuclear phagocytes [7,18]. Considering the enormous potential impact of exosome-mediated delivery of regulatory RNAs to either recipient leishmania or mammalian host cells or both, we sought to investigate whether leishmania exosomes carry RNA cargo. Here, we provide unambiguous evidence that leishmania parasites of two distinct species, namely *L. donovani* and *L. braziliensis*, release exosomes containing RNA sequences. These RNA sequences were heterogeneous, but overall short in length (25–250 nt). Thus, despite the abundance of longer sequences in total cell RNA, we were unable to detect them in exosomes. The enrichment of short RNA sequences in leishmania exosomes is concordant with the majority of reports on exosome RNA in other organisms published thus far (e.g. [22,36]). Although, there have been a few reports of the presence of longer RNAs such as full length ribosomal RNAs [39] and mRNAs [21] as well. The RNA cargo of exosomes is largely dependent on the cell of origin and appears to be affected by environmental conditions such as infection or nutritional stress [12], which likely explains the observed differences.

One important property of exosomes is their capacity to act as both short and long distance messengers. RNA-containing exosomes have been detected in a variety of human body fluids such as plasma, saliva and semen [40-42], which supports a role in long distance communication. As RNases are ubiquitously present in all organisms, RNAs travelling long distances need to be protected from degradation. In our *in vitro* experiments, we were able to show that leishmania exosomal RNA cargo is protected from degradation by exogenous RNase. When we incubated PMA-differentiated THP-1 cells *in vitro* with exosomes purified from axenic amastigotes of *L. donovani*, we saw that the exosome RNA cargo was readily taken up by host cells. This finding suggests that it should be possible for leishmania-derived RNAs to gain access to host cells through exosomes *in vivo*.

Numerous studies on exosome RNA have reported the presence of small regulatory RNAs such as micro RNAs (miRNAs) in these vesicles. It was of interest, therefore, to examine the leishmania exosome RNA content in detail by high throughput sequencing. It is important to mention here that leishmania are a special case with regard to small regulatory RNA pathways: *L. braziliensis* and other species from the new world *Leishmania (Viannia)* subgenus have been shown to have a functional RNAi pathway and actively produce siRNAs [24,32]. Conversely, this pathway appears to have been evolutionarily lost in old world *Leishmania (Leishmania)* species, such as *L. major* and *L. donovani* [24]. With this contrast in mind, we elected to sequence exosome RNA from *L. braziliensis* and *L. donovani* in parallel in order to compare the exosome RNA trascriptome of an RNAi-competent organism with an RNAi-deficient one.

When sequencing exosomal RNAs from these two leishmania species, we found that they both contained a variety of small non-coding RNA species, the majority of which appeared to be cleavage products derived from longer known non-coding RNAs such as rRNA, tRNA, snoRNA and snRNA. We also saw a small number of reads mapping to protein coding genes. In addition, we discovered a number of novel transcripts that were conserved in both libraries, and *L. braziliensis* exosomes uniquely contained transcripts derived from siRNA-coding putative mobile elements and repeats, such as SLACS and TATEs [32]. Other studies looking at mammalian exosome RNA content by deep sequencing have reported a similar composition of the exosomal transcriptome, with a dominant fraction of sequences being derived from rRNA and other non-coding RNA [36,37,39,43]. Conspicuously, sequences derived from protein coding genes seem to be underrepresented in exosomes. Thus, it appears that exosomes from many diverse organisms selectively package non-coding RNAs, the exact function of which still needs to be determined.

Importantly, our study is the first to purify *bona fide* exosomes from a protozoan parasite and provide a comprehensive analysis of high-throughput sequencing data of exosomal RNA cargo. By virtue of comparing two distinct (old and new world) species of leishmania, we have made the serendipitous discovery that the packaging of specific RNA sequences into exosomes appears to be a conserved phenomenon in leishmania. At the present time it remains unclear whether our findings are illustrative of what happens in other eukaryotic pathogens; however, there is some evidence indicating that the release of RNA within vesicles might occur in other parasitic organisms as well. In particular, there have been two articles published by independent groups that demonstrate the release of tRNA-derived small RNAs and other types of RNA within extracellular vesicles shed by the protozoan *T. cruzi* [17,44]; however, these vesicles were not characterized or classified as *bona fide* exosomes. The distinction between exosomes and other extracellular vesicles is important, as their origin, mechanism of biogenesis and thus loading of cargo differs substantially [45]. Three other articles have been published looking at the RNA cargo of exosomes released by parasitic pathogens; one of them a protozoan (*Trichomonas vaginalis*) and the other two helminths (*Heligmosomoides polygyrus* and *Dicrocoelium dendriticum*). However, all of these studies have significant limitations in their experimental design and RNA analysis when compared with the present study. The study on *T. vaginalis* only shows a size profile of RNA purified from exosomes measured by Bioanalyzer, but no sequencing data on exosomal RNA [22]. The article on *D. dendriticum* describes the analysis of exosomes by high-throughput sequencing; however, data analysis was focused on looking at micro RNA and does not report on other types of RNA in the exosomes [46]. Lastly, a very recent report on *H. polygyrus* reports sequencing data of libraries that have been generated with RNA obtained from parasite secretions and a vesicular fraction collected by ultracentrifugation, but not RNA from *bona fide* exosomes that were purified with specific exosome purification protocols [47]. The limitations of these studies do not allow for a direct comparison with our data and do not definitely answer the question whether the release of specific types of RNA within exosomes is a widespread phenomenon among parasites. While the data available suggest that this may certainly well be the case, further research will be needed to confidently answer this question.

Results from a number of studies [48-51] led to the suggestion that fragments derived from non-coding RNA species such as rRNA, snoRNA, vault RNA (vRNA) and tRNA can act as regulatory RNAs similar to miRNAs in RNAi. This hypothesis was based upon the finding that these fragments were shown to bind to Argonaute (AGO) proteins and formed RNA-induced silencing complexes (RISCs) which regulate expression of target mRNAs. *L. donovani* does not have the canonical proteins that are required for functional RNAi including AGO. However, an AGO/PIWI-like protein homolog, has been found in RNAi-deficient leishmania and other trypanosomes [52]. The function of this AGO homolog is currently unknown; however, one study suggested that it is not involved in the biogenesis or stability of siRNAs [53]. The presence of an alternative pathway of regulation of gene expression in RNAi-deficient trypanosomatids is likely, since these organisms perform their transcription polycistronically and hence regulation of expression of individual genes can only take place at the post-transcriptional level. A number of studies have indicated that post-transcriptonal regulation of gene expression in trypanosomes may involve *cis*-acting regulatory motifs within the 3′ UTRs of mRNAs and *trans*-acting RNA-binding proteins [54-56]. Other evidence for the presence of an alternative RNAi pathway in RNAi-deficient trypanosomatids comes from a recent series of studies in *T. cruzi*. The authors identified a unique AGO/PIWI protein termed TcPIWI-tryp that is expressed in all life cycle stages of the parasite and localizes to the cytoplasm [57]. Interestingly, they found that TcPIWI-tryp bound to a repertoire of RNAs distinct from siRNAs, namely small RNAs derived from rRNAs and tRNAs [23]. However, while these findings are intriguing, it remains to be established whether these complexes function in regulation of gene expression.

A large portion of reads in both our libraries mapped to rRNA genes in the reference genomes and they appeared to be shorter fragments. The presence of rRNA-derived sequences in leishmania exosomes is in agreement with other recent reports on exosome RNA cargo [39,58]. Sequences mapping to rRNAs have also been found in other types of extracellular vesicles, for example shed vesicles released by *T. cruzi* [17,44]. At this point it is unknown whether rRNA fragments have any specific function. Some limited evidence has been presented to show that rRNA fragments are produced by specific cleavage rather than random degradation in humans and mice [59]. These specific products were characterized by termini specific processing and asymmetric stabilization. However, in our data, no bias for either 5′ or 3′ processing was observed (Additional file 7: Table S4), but mapping of reads was rather scattered along the entire length of the rRNA gene. Moreover, we did not see enrichment of specific rRNA fragments derived from a subset of genes. Further study will be needed to elucidate whether rRNA fragments in leishmania exosomes are specifically enriched or selectively packaged.

Our finding that leishmania exosomes are overall enriched in non-coding RNA fragments which are taken up by host mononuclear cells, raises the interesting possibility that these RNA fragments may interfere with gene expression in the host, possibly by binding to host AGO. This type of epigenetic regulation of gene expression across kingdoms has been proposed, but so far little consistent and conclusive evidence has been presented. One elegant study recently showed that small RNAs from the plant fungal pathogen *Botrytis cinera* could silence Arabidopsis and tomato genes involved in plant immunity by binding host AGO [60]. This was the first time that naturally occurring cross-kingdom RNAi was shown to be a potential, novel virulence mechanism. Regarding human pathogens, some evidence has been presented that *T. cruzi* produces tRNA-derived small RNAs (tsRNAs) that may be delivered to susceptible mammalian cells [17]. Moreover, it was shown that transfection of host HeLa cells with synthetic *T. cruzi* tsRNAs can modify the expression of specific genes as assessed by microarray [61]. It remains to be established, how these tsRNA-induced changes of host gene expression were brought about and if this process involves hijacking of host RNAi pathways. In what follows below, we discuss potential roles of the non-coding, small RNA species found in leishmania exosomes which we believe are most likely to have regulatory functions, namely novel transcripts, siRNAs and tRNA-derived small RNAs.

### Novel transcripts

When examining reads that were less abundant in the libraries, we discovered 12 distinct genomic loci (Table 3 and Figure 4) that apparently gave rise to transcripts which have not been previously described. None of these transcripts had homology to any annotated gene in TriTrypDB or GenBank. BLAST search, however, confirmed that these non-annotated genomic sequences were conserved amongst most leishmania species. In considerable interest, we found that all of these novel transcripts had a spliced leader site upstream of their 5′ end (see Figure 4 for two examples) implying that they are processed alongside other transcripts during *trans*-splicing. The fact that the majority of novel transcripts contained one or more ORFs suggests that they may be translated into peptides or proteins. However, we were not able to find any homologous protein in any other species.

Two of the twelve novel transcripts that were most abundant from the group were further examined by quantifying their expression in exosomes in comparison to total cells by Northern analysis (Figure 4B). We found that both of these transcripts produced shorter processing products that were uniquely present in exosomes. This indicates that cleavage products of these transcripts may be specifically targeted for packaging into exosomes for release from the cell. The lack of a signal for these shorter products in Northerns of leishmania total RNA may also explain why these transcripts have not been reported in any of the previous studies on sequencing the leishmania transcriptome. Another possibility for why they have not previously been found is that they could be differentially expressed in the different life cycle stages (as we focussed only on axenic amastigotes).

One question that remains to be answered is what type of RNA these novel transcripts represent (protein coding, structural, regulatory) or whether they represent novel type(s) of RNA. Further studies will be needed to properly address the functions of these novel transcripts.

### siRNAs

We detected a number of sequences in the *L. braziliensis* exosome RNA library that mapped to siRNA coding loci such as SLACS and TATEs in the *L. braziliensis* genome. Even though the functional significance of endogenous siRNAs in *L. braziliensis* is still unclear, they are thought to be a genome defence mechanism to control the spread of potentially harmful nucleic acids, such as mobile elements, repeats and viruses [32]. Although these sequences were detectable in our library, they were generally in low abundance when compared to fragments of rRNA or tRNA. The fact that half of these sequences were each sense and antisense supported their tentative identity as siRNAs, given that one cardinal feature of siRNAs is that they are double stranded. Moreover, these were the only type of sequences in our libraries where this phenomenon was observed, as the vast majority of the other sequences (rRNA and tRNA fragments) were present only in the sense direction. The lengths of these putative siRNA sequences did not correspond exactly to what has been reported for *L. braziliensis* mature siRNAs (*Lbr*AGO1-bound) [32]. This might be due to different library construction strategies (size selection), it may be that what we detected were siRNA precursors, or that the sequences we detected were derived from distinct regions within the SLACS and TATEs loci. The finding that *L. braziliensis* packages these putative siRNA sequences as cargo of exosomes is of significant interest. It implies that these RNAs may function not only in the cell where they originated, but that they may also act in intercellular communication when taken up by other leishmania or by host cells or both. To our knowledge, no other parasite has been shown to release pathogen-derived siRNAs within vesicles directly. Further studies will be needed to confirm the identity of the sequences as siRNAs and delineate their function in parasite biology and in host-pathogen interaction.

### tRNA-derived small RNAs

A striking finding of the present study was the abundance of tRNA fragments principally originating from a small subset of tRNA isoacceptors (Figure 4B) that were highly conserved in the *L. donovani* and *L. braziliensis* exosome transcriptomes. Only recently tRNA fragmentation has been recognized as a specific process. tRNA fragments have been found in all domains of life and can be divided into several categories, depending on the cleavage site: cleavage within the anticodon loop gives rise to 5′ and 3′ tRNA halves (30–35 nt), and cleavage within the D-arm (5′) or T-arm (3′) gives rise to smaller tRNA-derived RNA-fragments (tRFs) (13–20 nt) [62]. Each of these fragments appears to be generated through distinct pathways. tRNA halves are known to be produced in response to stress [62], whereas the smaller tRFs, on the other hand, can be generated at any point of tRNA processing, by Dicer-dependent or –independent mechanisms [62,63]. Together, tRNA halves and tRFs are referred to as tRNA-derived small RNAs (tsRNAs). tsRNAs have recently been described in higher as well as lower eukaryotes Their physiological function is not well understood, but they have commanded increasing interest due to their suspected regulatory nature. Notably, it appears that tRNA halves and tRFs have distinct biological functions. In human cells, tRNA halves were found to inhibit protein translation by specifically targeting the translation initiation machinery and displacing elongation initiation factors [64]. tRFs, on the other hand, were shown to be involved in regulation of translation by directly binding to the small ribosomal subunit and interfering with peptidyl transferase activity in archae [65]. Furthermore, a similar mechanism was observed in human cells, where a tRF was shown to inhibit translation by affecting peptide bond formation [66]. In addition to these effects on translation, tRFs have also been shown to function in gene silencing. Haussecker *et al.* showed that tRFs can associate with Argonaute proteins, however, they associated more effeciently with the non-silencing AGO3 and AGO4 [50]. Furthermore, they saw that tRFs can affect the silencing activities of miRNAs and siRNAs, indicating a potential broad based role in regulating RNA silencing. In another study it was found that tRFs can function like miRNAs in RNAi and inhibit the expression of RPA1 (a protein involved in DNA repair) by binding to the 3′UTR of its mRNA [67].

A small number of studies have looked at the presence of tsRNAs in protozoan parasites. In *Plasmodium berghei* and *Toxoplasma gondii*, tRNA-halves were detected, and a relation between tRNA-half production and growth rate was observed [68]. However, the precise function of these parasite-derived tRNA-halves remains unknown. Interestingly, tsRNAs were recently discovered in leishmania’s close relative *T. cruzi*. Despite the fact that the lengths and origins of tsRNAs differed slightly from study to study, their production was convincingly demonstrated in both trypomastigotes and epimastigotes in a number of reports [28,29,69]. Importantly, it was found that *T. cruzi* tsRNAs were bound to TcPIWI-tryp, a distinct Argonaute protein that has been described in this RNAi-deficient organism [23,57]. The majority of these TcPIWI-tryp bound tsRNAs corresponded to the 5’ halves of tRNA-Glu. Importantly, TcPIWI-tryp-tsRNA complexes were also found in vesicles shed from *T. cruzi.* The authors proposed that these vesicles may have a role in life cycle transition from epimastigote to trypomastigote as well as contribute to infection susceptibility of mammalian cells [17]. These findings provide evidence that tsRNAs in *T. cruzi* may participate in non-canonical regulatory pathways and raise the question as to whether a similar phenomenon may be operative in leishmania.

In the present study, we provide evidence that leishmania also produces specific tsRNAs, and that these potential regulatory RNAs are released by the intracellular amastigote stage within *bona fide* exosomes, competent for delivery to mammalian cells. The major fraction of tsRNAs found in both *L. donovani* and *L. braziliensis* exosomes were 5′ tRNA halves, however, we also found shorter tsRNAs derived from the D-arm or T-arm of the tRNA, corresponding to 5′tRFs and 3′tRFs. The production and presence of tRNA halves in exosomes from leishmania amastigotes might be a result of the elevated temperature and acidic pH the parasites were exposed to. We found that the vast majority of tsRNAs in leishmania exosomes were derived from tRNA-Asp, tRNA-Gln, tRNA-Leu, tRNA-Glu and tRNA-Gly (Table 4 and Figure 5) and this was highly conserved between *L. donovani* and *L. braziliensis*. Although we did not carry out a comprehensive and quantitative analysis of the frequencies of all tsRNAs in leishmania whole cells, strikingly, we found by Northern blotting that tsRNAs from tRNA-Asp and tRNA-Leu were highly enriched in exosomes, with no detectable amounts in leishmania total RNA. This indicates that these tsRNAs are preferentially and quantitatively packaged into exosomes to be released from the cell rather than being retained in the whole cell (minus exosomal) RNA pool.

At this point, the mechanism of biogenesis and function of tsRNAs in leishmania is unknown. We are cannot, therefore, be 100% certain that the same classification of tRNA halves and tRFs as recently proposed by several groups [62,63,70] applies to our data. However, as many of the characteristics (length, cleavage site, isoacceptor origin) correspond to what has been reported in other organisms, we conclude that the phenomenon of specific tsRNA generation is evolutionarily conserved in leishmania as well. Based upon our initial functional predictions it appears clear that leishmania tsRNAs are not highly similar to canonical mammalian miRNAs or siRNAs. Further detailed investigations will be needed to delineate the functions of tsRNAs in leishmania biology, what roles they play in parasite-parasite, parasite-vector or parasite-host interactions, whether this involves their association with the host RNAi machinery and how they are targeted for exosomal packaging.

## Conclusions

In summary, this report provides evidence that leishmania exosomes are enriched in short sequences derived from non-coding RNAs such as rRNAs and tRNAs. Moreover, exosomes contain a number of novel transcripts, albeit in relatively low abundance. The RNAi-proficient *L. braziliensis* appears to package putative siRNAs or their precursors into exosomes, whereas RNAi deficient *L. donovani* does not. Based on Northern analyses, our data indicate that specific RNA sequences are selectively, and in some cases quantitatively packaged into exosomes. This conclusion is supported further by the highly biased distribution of sequences detected in exosomes over only a subset of genes in the leishmania genome combined with a striking paucity of transcripts derived from protein coding genes which are otherwise abundant in total cellular RNA.

Importantly, our findings provide at least three lines of evidence arguing for the presence of an evolutionarily conserved mechanism for packaging small non-coding RNAs into exosomes in leishmania: 1) the high degree overlap between the top 20 most abundant sequences found in *L. donovani* and *L. braziliensis* exosomes, 2) the vast majority of identified novel transcripts was present in exosomes from both species, and 3) the most abundant tsRNAs found in exosomes were derived from a highly biased subset of the same tRNA isoacceptors in both species. Taken together, the data argue strongly that leishmania exosomal RNA sequences are specifically produced and packaged into exosomes for release, likely with the purpose of modifying host cell phenotype to support chronic infection. The investigation of the precise functions of these small, non-coding, leishmania RNAs should contribute significantly to our understanding of parasite biology and mechanisms of pathogenesis.

## Methods

### Cell culture

*L. donovani* Sudan strain S2 promastigotes were routinely cultured in M199 (Sigma-Aldrich) with 10% heat inactivated fetal bovine serum (FBS, Gibco), 20 mM HEPES (Stemcell), 6 μg/mL hemin (Sigma-Aldrich), 10 μg/mL folic acid (Sigma-Aldrich), 2 mM L-glutamine (Stemcell), 100 U/mL penicillin/streptomycin (Stemcell) and 100 uM adenosine (Sigma-Aldrich) at 26°C. Every 3 days the organisms were subcultured 1:10 in fresh medium and were kept in culture for a maximum of 20–25 passages. Fresh parasites were obtained by purification of amastigotes from spleens of infected Syrian Golden hamsters followed by *in vitro* transformation into promastigotes by culturing for 7 days at 26°C in promastigote media.

*L. braziliensis* (clinical isolate from the Peruvian Amazon region) promastigotes were routinely cultured in the same media as above except for supplementation with 20% FBS. *L. braziliensis* promastigotes were subcultured 1:5 every 3 days in fresh media and kept at 26°C.

### Purification of exosomes

Exosomes were purified from *L. donovani* and *L. braziliensis* axenic amastigote culture supernatant as described previously [7,18]. Briefly, 400–800 mL of day 5 promastigotes (at a concentration of 5×10E7 cells/mL) were washed 2× with Hank’s buffered salt solution (HBSS, Sigma-Aldrich) followed by incubation in serum-free buffered exosome collection media at pH = 5.5, RPMI1640 supplemented with 1% D-glucose, 20 mM HEPES, 2 mM L-glutamine, 100 U/mL penicillin/streptomycin and 25 mM MES (all from Sigma-Aldrich), at 34°C for *L. braziliensis* and 37°C for *L. donovani*. After 24 hours of incubation, exosomes were purified from the 400–800 mL culture supernatant under endotoxin-free conditions by a series of centrifugation and filtration steps, followed by flotation on a sucrose cushion, as described in [18]. After a final pelleting step at 100,000×g for 1 hour, purified exosomes were resuspended in 50–100 μL of PBS and processed immediately (in case of RNA extractions) or stored at 4°C for a maximum of 5 days (for macrophage uptake experiment and Nanosight analysis).

### Nanosight particle tracking analysis

The size and concentration of the isolated exosomes were analysed using the NanoSight™ LM10-HS10 system (Malvern Instruments). For analysis, a monochromatic laser beam (405 nm) was applied to the diluted exosome solution (1:100 in 0.02 μm filtered PBS) that was injected into a LM12 viewing unit using a computer controlled syringe pump. NanoSight™ tracking analysis (NTA) software version 2.3 was used to produce the mean and median vesicle size together with an estimate of particle concentration. Samples were measured 3 times to confirm reproducibility.

### Extraction and biochemical characterization of RNA

RNA was purified from leishmania exosomes by phenol/chloroform extraction using all RNA-grade reagents. For this purpose, 150 uL of LETS buffer (0.1 M LiCl, 0.01 M Na_2_EDTA, 0.01 M Tris-Cl pH = 7.4, 0.2% SDS, all Sigma-Aldrich) was added to 50 μL of exosomes resuspended in PBS followed by addition of 200 μL Ultra-Pure buffer-saturated phenol pH = 7.4 (Life Technologies). The mixture was vortexed vigorously and centrifuged for 2 minutes at 13,000×g in a microcentrifuge at room temperature. The upper aqueous phase was collected and the phenol extraction was repeated once more followed by two extractions over 200 μL chloroform each (Fisher Scientific). RNA was precipitated by addition of 0.3 M NaCl, 2 μg/mL glycogen (Ambion) and 75% EtOH, and incubation at −20°C over night. RNA was pelleted by centrifugation at 13,000×g for 30 minutes at 4°C. RNA pellets were washed with ice-cold 75% ethanol and resuspended in 10–20 μL ddH_2_O. RNA concentration was determined by measuring the OD260 with the nanodrop (Thermo).

To look at length profiles of exosome-derived RNA, 2 μg of purified leishmania total RNA and 1 μg of exosome RNA were first treated with 5 units DNase I (Thermo) to remove potential DNA contamination. After incubation for 30 minutes at 37°C, DNase was inactivated by addition of 2.5 mM EDTA and incubation at 65°C for 10 minutes followed by phenol-chloroform extraction and ethanol precipitation as above. RNA was resuspended in 4 μL ddH_2_O and RNA length profiles were obtained with the Agilent Bioanalyzer using the RNA 6000 Pico kit according to the manufacturer’s instructions (Agilent). Alternatively, DNase-treated RNA was run on a 15% polyacrylamide gel, stained with SYBR green (Life Technologies) and imaged with UV-imaging.

To confirm identity of nucleic acid purified from exosomes as RNA, 1–2 μg of phenol/chloroform extracted RNA was treated with DNase (as above), followed by treatment with either 0.4 mg/mL RNase A (Thermo) for 15 mins at 37°C or hydrolysis with 50 mM KOH (Sigma-Aldrich) for 15 min at 95°C. Samples were then 5′ end labeled according to the manufacturer’s instructions using polynucleotide kinase (PNK) (New England Biolabs, NEB) and γ^32^PdATP (Life Technologies) and run on 15% polyacrylamide gels followed by imaging with a Typhoon phosphor-imager (GE Healthcare).

To assess whether the exosomal membrane was protecting the vesicular RNA content from degradation by exogenous RNases, intact exosomes resuspended in PBS (from 400 mL culture supernatant, split into 4 samples) were treated with 0.4 mg/mL RNAse A for 15 mins at 37°C in the presence or absence of 0.1% Triton X-100 (Sigma-Aldrich). As a control for RNase activity, 1 μL of prepared RNA pico ladder (Agilent) was treated with RNase A under the same conditions. After incubation, samples were extracted with phenol/chloroform 2× each and RNA was precipitated with ethanol as above. Samples were then treated with DNase, again phenol/chloroform extracted and ethanol precipitated, resuspended in 4 μL ddH_2_O and run on the Agilent Bioanalyzer to determine whether or not RNA had undergone degradation.

### Vesicle delivery of RNA cargo to macrophages

Exosomes were purified from 400 mL culture supernatant of *L. donovani* axenic amastigotes as described above. Pelleted exosomes were resuspended in 100 μL PBS. Protein concentration in the exosome preparation was determined using the Micro BCA Protein Assay kit (Pierce). Exosomes were then stained with the membrane-permeant, RNA-specific dye SYTO RNASelect (Life Technologies) according to the manufacturer’s recommendations. For this purpose, the SYTO dye was diluted in DMSO and added to the resuspended exosomes at a final concentration of 10 μM, followed by 20 minutes incubation at 37°C. Excess unbound dye was removed by washing twice with 1 mL PBS, pelleting the exosomes at 100,000×g for 1 hour at 4°C. Exosomes were then resuspended in the original volume of PBS (100 μL). Labelling efficiency was assessed by fluorescence microscopy using an Axioplan II epifluorescence microscope equipped with 63×/1.4 Plan-Apochromat objective (Carl Zeiss Inc). Images were recorded using an AxioCam MRm Camera coupled to the AxioVision software Version 4.8.2 (Carl Zeiss Inc.).

To investigate the exosome-mediated delivery of RNA to host macrophages, THP-1 cells were differentiated over night with 10 ng/mL phorbol-12-myristate 13-acetate (PMA), followed by washing and resting cells for 24 hours. Differentiated cells were then treated with labeled exosomes for 2 hours at 37°C. As a negative control, cells were treated with labelled exosomes and incubated at 4°C for 2 hours, preventing phagocytosis. For quantification of exosome RNA uptake, exosome-treated THP-1 cells were washed 3× with PBS to remove non-internalized exosomes. Cells were then fixed with 2% paraformaldehyde (Sigma) in PBS for 15 minutes at room temperature. After fixation, cells were again washed with PBS and then analyzed by flow cytometry (FACS Calibur, BD). To verify that exosomes were in fact internalized and not just bound to the cell membrane, the same experiment was performed with THP-1 cells grown on coverslips to be analysed by confocal microscopy. After incubation, cells on coverslips were washed and fixed as above, permabilized with 0.1% Triton X-100 in PBS for 5 minutes, and stained with Alexa Fluor 594 phalloidin (Life Technologies) for 1 hour at room temperature in the dark. After 3× washing with PBS, coverslips were mounted with Prolong Gold antifade mounting media containing DAPI (Life Technologies) to detect macrophage nuclei. Confocal microscopy was done with a Leica DMIRE2 inverted microscope equipped with a SP2 AOBS laser scanning head. This is a filter-free spectral confocal and multiphoton microscope, and all imaging operations which include selections of laser, detection channels and other functions are fully automated and computer controlled. Pictures were taken with a 63× magnification oil immersion objective.

### Library construction and sequencing

We used 1–2 μg of RNA extracted by phenol/chloroform extraction from *L. donovani* and *L. braziliensis* exosomes (from one individual exosome preparation each, from 800 mL supernatant) as starting material. RNA was first treated with DNase I (as described above) to remove potentially contaminating DNA. To remove 5′ phosphates on the RNA, we first performed a calf intestinal alkaline phosphatase (CIP) treatment using 1 unit of CIP (Roche) per 10 μL reaction and incubation for one hour at 37°C. Once incubation was completed, samples were phenol-chloroform extracted twice and ethanol precipitated as described above. In order to monitor the efficiency of the CIP treatment, a parallel reaction was spiked with a 24 nt long radiolabelled RNA, and pre and post incubation with CIP were loaded in a 20% denaturing polyacrylamide gel to follow the disappearance of counts. Next, the CIP treated RNA sample (resuspended in 10 μL ddH2O) was treated with tobacco acid phosphatase (TAP) to remove 5′ caps. Half of the CIP treated RNA sample was combined with 2.5 units of TAP (Epicentre), 1X TAP buffer, brought to a final volume of 10 μL with ddH_2_O and incubated for one hour at 37°C. In order to control for the efficiency of 5′ cap removal, a parallel reaction was spiked with γ^32^PdATP and pre-and post ligation samples were loaded in a 20% plyacrylamide gel to monitor the disappearance of counts. RNA was ethanol-precipitated and resuspended in 10 μL ddH_2_O. The CIP and TAP treated RNA were then labeled with polynucleotide kinase (PNK) to have the same 5′ phosphate in all RNA molecules about to be ligated. Ten units of PNK (NEB) were used along with 1X PNK buffer (NEB), and γ^32^PdATP in a 10 μL reaction and incubation for one hour at 37°C. Next, ~ 10% of the sample was loaded onto a denaturing 15% polyacrylamide gel. The remainder of the PNK reaction was taken to a 30 μL volume with ddH_2_O and run through a dye terminator removal (DTR) cartridge (EdgeBio) following manufacture’s indications in order to remove ions and unincorporated γ^32^PdATP. The sample was ethanol precipitated and resuspended in 5 μL of ddH_2_O. The next step was to ligate a custom adenylated AppDNA adaptor (5′ App-GAAGAGCCTACGACGA) to the 3′ end of RNA molecules. This adaptor was slightly modified so that the 3′ end was blocked in order to prevent self ligation. Half of the pre-treated exosomal RNA sample was combined with T4 RNA ligase buffer (50 mM HEPES, pH 8.3, 10 mM MgCl2, 3.3 mM DTT, 10 g/ml BSA and 8.3% glycerol; [71], 2.5 U of T4 RNA Ligase (Epicentre), and 20 μM AppDNA adaptor. Reactions were incubated at room temperature for one hour, and then the enzyme was denatured by heating at 65°C for 20 minutes. Samples were gel purified on 10% polyacrylamide gels to remove un-ligated adaptor. A second ligation reaction was set up to attach an RNA adaptor (5′ rAUCGUAGGCACCUGAAA) to the 5′ end of the RNA-DNA hybrid. Conditions were the same as described above for the first ligation reaction with the only difference being that γ^32^PdATP (final concentration of 0.4 mM) was added. The ligation reaction was gel purified from a denaturing 10% polyacrylamide gel and the recovered material was used as a template in a reverse transcription (RT) reaction. For this purpose, half of the recovered sample was combined with 100 μM RT primer (5′ TCGTCGTAGGCTCTTC), ddH_2_O and incubated at 80°C for two minutes. After cooling samples down slowly, 1X First Strand Buffer (Life Technologies), 0.8 μM dNTP and 200 U of Superscript II Reverse Transcriptase (Life Technologies) were added. Controls with no enzyme and no template in the reaction were prepared in parallel. Reactions were incubated for 1 hour at 48°C. The RNA template was hydrolyzed by heating in the presence of 100 mM KOH followed by neutralization with 1 M Tris–HCl pH = 5 (to a final pH = 8), and the resulting cDNA was isolated on a 10% denaturing polyacrylamide gel. 20 cycles of PCR amplification were performed in the presence of 5 mM MgCl_2_, 100 μM dNTPs, 1 μM each forward primer (5′ ATCGTAGGCACCTGAAA) and reverse primer (same as RT primer), 1X Taq buffer and 2.5 Units Taq polymerase (UBI). PCR products were then gel purified and quantified by Qubit (Life Technologies) and used as input material for ligation of TruSeq adapters (Illumina) according to the manufacturer’s recommendations. 150 base pair, paired-end sequencing was performed using an Illumina MiSeq instrument (Illumina) at the Epigenomics core of Albert Einstein College of Medicine, NY.

### Sequencing data analysis

After completion of Illumina paired-end sequencing and read quality control checking by FastQC (http://www.bioinformatics.babraham.ac.uk/projects/fastqc/), both *L. donovani* and *L. braziliensis* exosomal RNA reads had their adapters trimmed by cutadapt version 1.0 (http://journal.embnet.org/index.php/embnetjournal/article/view/200/479). For each library, the output files from the trimming were separated into RNA adapter-trimmed reads and DNA adapter-trimmed reads, and the former was used to guide the assignment of correct orientation for all reads sequenced. We ran FLASh (settings: −M 100 –x 0.2) [72] and FASTX-Collapser (http://hannonlab.cshl.edu/fastx_toolkit) to respectively combine the mates, for the cases where DNA inserts were shorter than twice the length of reads, and then collapsed identical sequences into single ones to facilitate handling the data in subsequent specific analyses. Bowtie2 version 2.1.0 (settings: very-sensitive-local –N1) [73] was used to align the collapsed reads (cReads) from both libraries against their respective reference genomes (LdBPK (*Leishmania donovani* strain BPK282A1) and LbrM (*Leishmania braziliensis MHOM/BR/75/M2904*) from TriTrypDB version 6.0), as well as against the species with the best assembled and annotated genome (LmjF, *L. major* MHOM/IL/81/Friedlin*,* TriTrypDB version 6.0). The very-sensitive-local setting of Bowtie 2 uses a seed length of 20 nt for the alignment, and the –N1 command allows for only one mismatch on that seed alignment. The alignments with LdBPK and LbrM were used to categorize the exosomal RNAs for the respective species, relying on htseq-count script [74] and the GFF files provided by TriTrypDB v6.0. The alignment with LmjF was done mainly to refine the analyses of reads mapping onto tRNAs. Of note, right after bowtie2 execution, samtools version 0.1.18 [75] was applied to generate sorted bam files, which were then used as input to cufflinks (settings: −u --min-intron-length 3 --3-overhang-tolerance 25 --overlap-radius 10 --min-frags-per-transfrag 1) [76] for the assembly of reads mapping on the same locus into individual “transcripts” or clusters. Artemis genome browser software [27] was used to manually inspect in greater detail and visualize the alignment of exosomal sequences with the reference genomes.

As mentioned above, we used reads mapping to *L. major* tRNAs for a better categorization of potential tRNA-derived small RNAs present within the exosomes. An ad-hoc PERL script was written to calculate the cReads position within each tRNA feature they mapped onto: 5′end (cReads mapping entirely on the 5′end half of the tRNA gene), mid-5′ (cReads starting on the first 1/3 and ending before the last 1/3 of the tRNA gene length), 3′end (cReads mapping entirely on the 3′end half of the tRNA gene), mid-3′end (cReads starting after the first 1/3 and ending within the last 1/3 of tRNA gene length), mid (cRead overlaps both halves of the tRNA gene and not within the 1/3 extremity regions). The same method was used to calculate the cReads position within each rRNA feature for rRNA fragments found in exosomes.

The discovered 12 novel transcribed loci had their nucleotide sequences translated by the getorf program from the EMBOSS package [77] with the following paramaters: −minsize 33 -find 1 –noreverse, which sets a 10 amino acids minimum ORF length, translates solely from ATG to STOP codons and only on the three possible frames from the same strand where the exosome RNA reads mapped to, respectively. In order to check whether the putative ORFs outputted by the getorf program have similarity to any already known protein, we ran sensitive blastp against nr-NCBI (−word_size 2 -num_descriptions 5 -num_alignments 5 -evalue 1e-3) and no hits were found for any of them.

To determine whether there were transposable elements-derived RNA fragments within leishmania exosomes and also discard any possibility of cross-contamination between the libraries, we performed a BlastN search [78] for *L. braziliensis*-specific SLACS/TATEs elements (extracted from TriTrypDB-6.0_LbraziliensisMHOMBR75M2904_ AnnotatedTranscripts.fasta downloadable file at tritrypdb.org). The following thresholds were applied during this screen: e-Value < = 1, identity > = 80% and query (cRead) coverage > = 70%.

To search for any sequences homologous to mammalian miRNAs within the leishmania exosomal RNA libraries, we ran blastn from the BLAST Plus package [79] version 2.2.29+ querying the top thousand most abundant cReads on each library against the whole human and mouse miRNA dataset (hairpin and mature) available at miRBase (mirbase.org). The blastn + parameters were the following: −dust no -word_size 4 -evalue 1 -outfmt 6, and we also established a cutoff of 70% identity and 70% sequence coverage (ad-hoc PERL script). In a parallel approach to identify host genes that could potentially be targeted by putative regulatory RNAs in leishmania exosomes, we aligned the cReads from both libraries against human (hg19, NCBI) and vector (*Lutzomyia longipalpis* and *Phlebotomus papatasi,*https://www.vectorbase.org/) reference genomes. Bowtie2 version 2.1.0 (settings: −-very-sensitive-local –N1) [73] and htseq-count script (http://www-huber.embl.de/users/anders/HTSeq/doc/overview.html) (using the option –s reverse, which reports reads mapping to annotated features on a reverse complement fashion) were used for this purpose.

### Northern blotting

Aliquots of ~3 μg RNA per lane (*L. donovani* axenic amastigote total RNA from a single culture, or exosome RNA pooled from 4 separate exosome preparations) were loaded onto 8% denaturing polyacrylamide gels. Gels were stained with SYBR Green and visualized, then the samples were blotted onto Hybond N^+^ nylon membrane (GE Healthcare). The membranes were UV cross-linked using a Stratalinker (1200 μJ for 30 seconds), blocked, probed and washed according to [80]. Twenty one and 150 nt long *in vitro* transcribed RNAs were used as size markers. For hybridization, 5′end labelled DNA probes were used (LdBPK_291610_leftof probe 5′ AAGGCGTCCCCATGATAACG, LdBPK_301180_leftof probe 5′ GACCTCAAGTATCTACGGGAGA, tRNA-Asp probe ASP1 5′ GGCGGGTATACTAACCACTATAC, tRNA-Leu probe LEU1 5′ AGACCACTCGACCATCTCA, tRNA-Leu probe LEU2 5′ TGGAACCTTAATCCAACGTCTT, 5.8S rRNA probe sequence was taken from [81]). For 5′ end labelling, the PNK labelling reaction was carried out as suggested by the manufacturer (NEB). The efficiency of γ^32^PdATP incorporation was determined by running a small fraction of the PNK reaction on a native 20% polyacrylamide/urea gel and typically ranged between 80-95%, resulting in probes with high specific activity. The blotted membranes were placed in glass bottles containing a minimal amount of hybridization buffer (6X SSPE, 1% SDS, 2X Denhart’s solution, 100 μg/mL of salmon sperm DNA; [82]) in a Hybaid™ mini oven MKII and pre-hybridized with constant rotation for 4 hours at 37°C. After pre-hybridization, approximately 10 μCi of labelled probe was added and the membrane was hybridized for at least 18 hours at 37°C. The next day, the membrane was washed twice with a high stringency solution (2X SSPE and 0.1% SDS), and twice with a low stringency solution (0.2X SSPE and 0.1% SDS) for 15 minutes each at room temperature. The radioactive signal from the membranes was detected using a Storm 820 phosphorimager. Quantification of signals was performed in Imagequant.

### Statistical analysis and graphs

R environment version 3.0 was used to generate read length distribution histograms for each library (calculating their mean and median values), as well as to perform the Pearson’s Correlation analysis regarding the tRNA-derived small RNA reads abundance and the amino acid usage frequency of the respective predicted proteomes. Other graphs were generated with GraphPad Prism 4.0 and EXCEL.

## Additional files

Additional file 1: Figure S1.
*Leishmania braziliensis* exosomes contain RNA. Exosomes were purified from *L. braziliensis* axenic amastigote culture supernatant as described in the Methods. RNA was extracted from exosomes with phenol-chloroform and then analyzed. A. Agilent Bioanalyzer RNA length profiles of exosome RNA alongside total RNA, B. RNA inside exosomes is resistant to degradation. Prior to RNA extraction, intact exosomes were left untreated or treated with either RNase A or TritonX-100 or both. Samples were then subjected to RNA extraction and run on the Agilent Bioanalyzer. Arrowhead indicates internal 25 nt marker. nt = nucleotides. Additional file 2: Figure S2.
*Leishmania donovani* exosomes can be efficiently stained with green fluorescent SYTO RNASelect dye. Exosomes were purified from 400 mL supernatant of *L. donovani* axenic amastigotes and stained with a membrane permeant, green fluorescent RNA-specific dye (as described in Methods). A sample each of stained and unstained *L. donovani* exosomes were then examined by microscopy using an Axioplan II epifluorescence microscope equipped with 63×/1.4 Plan-Apochromat objective (Carl Zeiss Inc). Images were recorded using an AxioCam MRm Camera coupled to the AxioVision software Version 4.8.2 (Carl Zeiss Inc.). Additional file 3: Table S1. Sequences found in *L. donovani* and *L. braziliensis* exosomes. File 1 ‘LD_Collapsed_reads.tab’: Collapsed Read IDs including full nucleotide sequences, File 2 ‘LD_vsLdoB-bowtie2-HTseq.tab’: Results of the Bowtie2 alignment of the *L. donovani* exosome sequencing library against the LdBPK reference genome, File 3 ‘LD_vsLmjF-bowtie2-HTseq.tab’: Results of the Bowtie2 alignment of the *L. donovani* exosome sequencing library against the LmjF reference genome. File 4 ‘LB_Collapsed_reads.tab’: Collapsed Reads including full nucleotide sequences, File 5 ‘LB_vsLbrM-bowtie2-HTseq.tab’: Results of the Bowtie2 alignment of the *L. braziliensis* exosome sequencing library against the LbrM reference genome, File 6 ‘LB_vsLmjF-bowtie2-HTseq.tab’: Results of the Bowtie2 alignment of the *L. braziliensis* exosome sequencing library against the LmjF reference genome. “cRead ID” refers to the identifier of the collapsed read, which is composed of the cRead number, followed by the number of copies. “Strand” refers to the strand the reads were aligning with, plus = top strand, minus = bottom strand. Additional file 4: Table S2. Other hits found in *L. donovani* and *L. braziliensis* exosomes. File 1 ‘LD_blastN-results-vsNT.tab’: Results of the blastN search of the unaligned reads (i.e. not aligned to LdoB or LmjF) from the *L. donovani* exosome sequencing library against the NCBI nucleotide collection database. File 2 ‘LD_GBhits_description.tab’: GenBank description of hits. File 3 ‘LD_reads_per_species.tab’: Summary of reads per species. File 4 ‘LB_blastN-results-vsNT.tab’: Results of the blastN search of the unaligned reads (i.e. not aligned to LbrM or LmjF) from the *L. braziliensis* exosome sequencing library against the NCBI nucleotide collection database. File 5 ‘LB_GBhits_description.tab’: GenBank description of hits. File 6 ‘LB_reads_per_species.tab’: Summary of reads per species. Additional file 5: Table S3. Overall alignment statistics. Additional file 6: Figure S3. Length histograms of reads mapping to rRNA genes. Additional file 7: Table S4. Mapping of reads to rRNA genes based on Bowtie 2 alignments with the LmjF reference genome. Additional file 8: Table S5. Putative ORFs found in the novel transcripts. Additional file 9: Table S6A. Results of blast search of all reads from *L. donovani* exosome library against human and vector genomes. Table S6B Results of blast search of all reads from *L. braziliensis* exosome library against human and vector genomes. Additional file 10: Table S7. Results of blast search of all reads from *L. donovani* and *L. braziliensis* exosome library against SLACS and TATEs database. Additional file 11: Table S8. Hits against miRbase: Top 1000 most abundant reads in both libraries were blast searched against human and mouse miRNAs.

## Abbreviations

AGO: Argonaute
BLAST: Basic local alignment search tool
cDNA: Complementary DNA
CDS: (protein) coding sequence
CIP: Calf intestinal phosphatase
CIR74: Chromosomal internal repeats, 74-nucleotide long
DNA: Deoxyribonucleic acid
FBS: Fetal bovine serum
HBSS: Hank’s buffered salt solution
LINE: Long interspersed elements
miRNA: micro RNA
mRNA: Messenger RNA
nt: Nucleotide
PAGE: Polyacrylamide gel electrophoresis
PBS: Phosphate buffered saline
PNK: Polynucleotide kinase
RNA: Ribonucleic acid
RNAi: RNA interference
rRNA: Ribosomal RNA
RT: Reverse transcription
scRNA: Small cytoplasmic RNA
SINE: Short interspersed elements
siRNA: Small interfering RNA
SL: Spliced leader
SLACS: Spliced leader-associated conserved sequence
snoRNA: Small nucleolar RNA
snRNA: Small nuclear RNA
srpRNA: Signal recognition particle RNA
TAP: Tobacco acid phosphatase
TAS: Telomere-associated sequence
TATE: Telomere-associated transposable element
tRF: tRNA-derived RNA fragment
tRNA: Transfer RNA
tsRNA: tRNA-derived small RNAvRNA, vault RNA
U: Unit
UTR: Untranslated region
