# Supplementary figures and images for: Small RNAs derived from tRNAs and rRNAs are highly enriched in exosomes from both old and new world Leishmania providing evidence for conserved exosomal RNA Packaging
Source: BMC Genomics. 2015 Mar 5;16(1):151. doi: 10.1186/s12864-015-1260-7 (PMC4352550; doi:10.1186/s12864-015-1260-7)

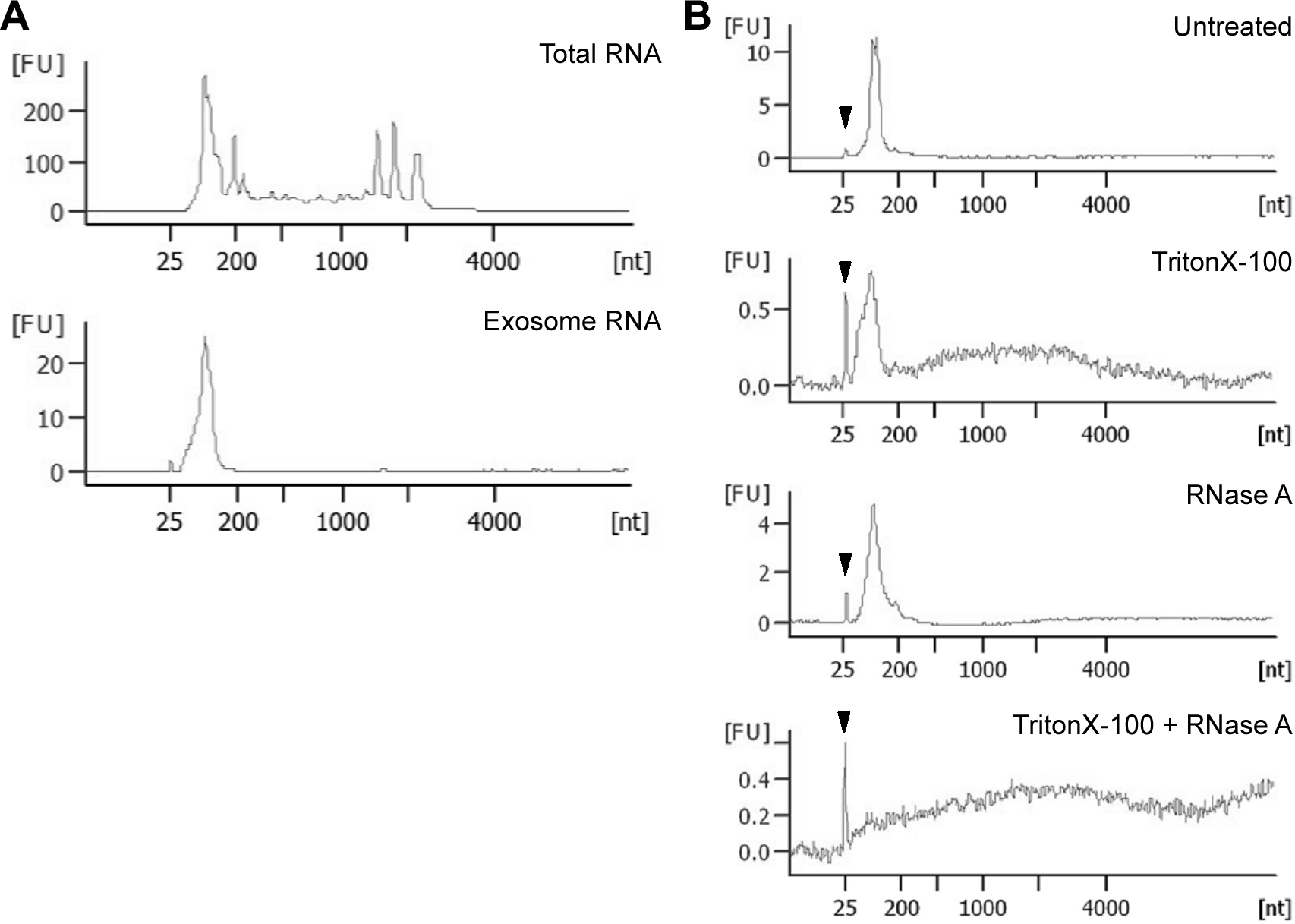

Supplement: Additional file 1: Figure S1. — Leishmania braziliensis exosomes contain RNA. Exosomes were purified from L. braziliensis axenic amastigote culture supernatant as described in the Methods. RNA was extracted from exosomes with phenol-chloroform and then analyzed. A. Agilent Bioanalyzer RNA length profiles of exosome RNA alongside total RNA, B. RNA inside exosomes is resistant to degradation. Prior to RNA extraction, intact exosomes were left untreated or treated with either RNase A or TritonX-100 or both. Samples were then subjected to RNA extraction and run on the Agilent Bioanalyzer. Arrowhead indicates internal 25 nt marker. nt = nucleotides. [file 12864_2015_1260_MOESM1_ESM.tiff]

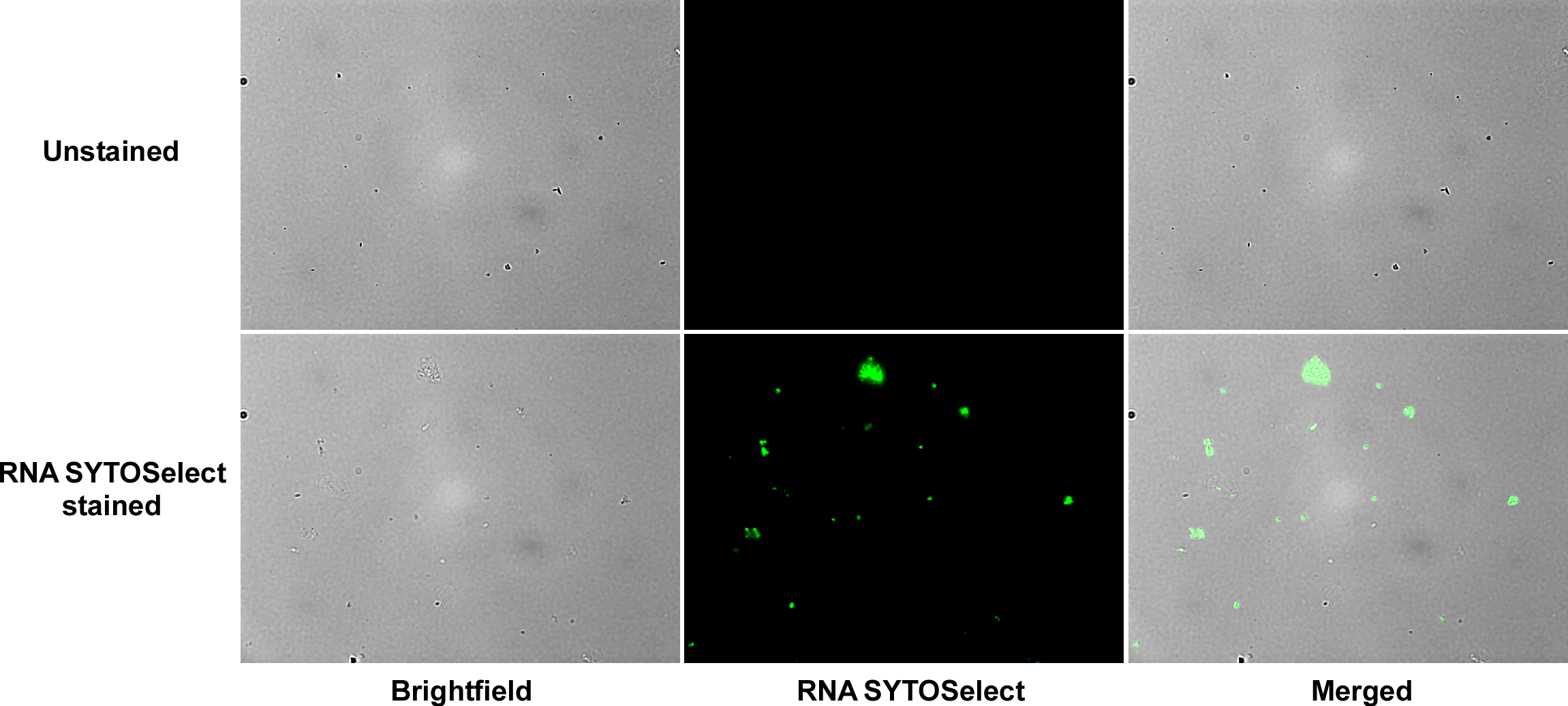

Supplement: Additional file 2: Figure S2. — Leishmania donovani exosomes can be efficiently stained with green fluorescent SYTO RNASelect dye. Exosomes were purified from 400 mL supernatant of L. donovani axenic amastigotes and stained with a membrane permeant, green fluorescent RNA-specific dye (as described in Methods). A sample each of stained and unstained L. donovani exosomes were then examined by microscopy using an Axioplan II epifluorescence microscope equipped with 63×/1.4 Plan-Apochromat objective (Carl Zeiss Inc). Images were recorded using an AxioCam MRm Camera coupled to the AxioVision software Version 4.8.2 (Carl Zeiss Inc.). [file 12864_2015_1260_MOESM2_ESM.tiff]

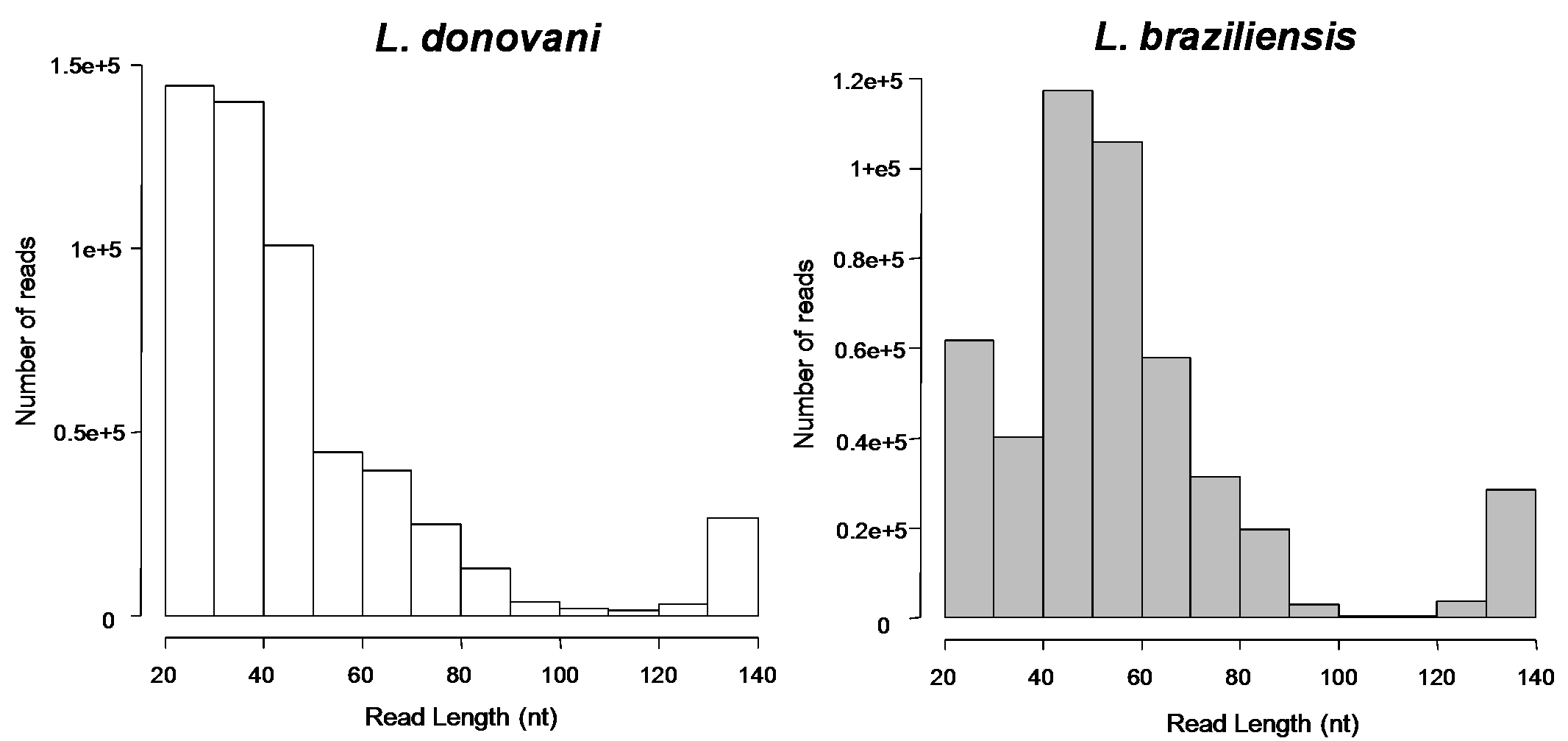

Supplement: Additional file 6: Figure S3. — Length histograms of reads mapping to rRNA genes. [file 12864_2015_1260_MOESM6_ESM.tiff]
