# Supplementary material for: Small RNAs derived from tRNAs and rRNAs are highly enriched in exosomes from both old and new world Leishmania providing evidence for conserved exosomal RNA Packaging
Source: BMC Genomics. 2015 Mar 5;16(1):151. doi: 10.1186/s12864-015-1260-7 (PMC4352550; doi:10.1186/s12864-015-1260-7)
Supplement: Additional file 5: Table S3. — Overall alignment statistics. [file 12864_2015_1260_MOESM5_ESM.docx]

**Supplementary Table 3. Sequencing and alignment results**

|  | ***L. donovani* library** | ***L. braziliensis* library** |
| --- | --- | --- |
| Total pairs of reads | 1435277 | 1062571 |
| Combined reads (mate 1 and mate 2 match) | 1196156 | 912820 |
| Uncombined reads (mates don’t match) | 239121 | 149751 |
| Total reads used for collapsing (combined reads plus (uncombined reads x2)) | **1674398** | **1212322** |
| Collapsed reads (cReads) | 688524 | 538034 |
| Unique and single copy | 574049 | 421086 |
| Alignment against respective reference genome  LdoB, LbrM [cReads] | 981412 (58.61%)  [243745] | 277363 (22.87%)  [89414] |
| Alignment against LmjF reference genome  [cReads] | 966213 (57.7%)  [234032] | 641046 (52.88%)  [200014] |
| Unaligned reads (LdoB for L. donovani library, LmjF for L. braziliensis library)  [cReads] | 692986 (41.4%)  [444779] | 571276 (47.1%)  [338020] |
| Unaligned reads showing hits on NT-NCBI  (no e-value cutoff result) | 471103 (28.1%)  [235745] | 440511 (36.3%)  [214674] |
| Amongst unaligned reads showing hits on NT-NCBI: reads mapping to other leishmania genomes | 4.93% | 4.17% |
| Total percentage of reads mapping to leishmania genomes | 63.54% | 57.01% |
| Reads that did not align to anything at all | 13.3% | 10.8% |
